# Extracellular inhibitors can attenuate tumorigenic Wnt pathway activity in adenomatous polyposis coli mutants: Predictions of a validated mathematical model

**DOI:** 10.1371/journal.pone.0179888

**Published:** 2017-07-14

**Authors:** Gili Hochman, Karin Halevi-Tobias, Yuri Kogan, Zvia Agur

**Affiliations:** Institute for Medical BioMathematics, Bene Ataroth, Israel; University of Washington, UNITED STATES

## Abstract

**Background:**

Despite considerable investigational efforts, no method to overcome the pathogenesis caused by loss of function (LoF) mutations in tumor suppressor genes has been successfully translated to the clinic. The most frequent LoF mutation in human cancers is Adenomatous polyposis coli (APC), causing aberrant activation of the Wnt pathway. In nearly all colon cancer tumors, the APC protein is truncated, but still retains partial binding abilities.

**Objective & methods:**

Here, we tested the hypothesis that extracellular inhibitors of the Wnt pathway, although acting upstream of the APC mutation, can restore normal levels of pathway activity in colon cancer cells. To this end, we developed and simulated a mathematical model for the Wnt pathway in different APC mutants, with or without the effects of the extracellular inhibitors, Secreted Frizzled-Related Protein1 (sFRP1) and Dickhopf1 (Dkk1). We compared our model predictions to experimental data in the literature.

**Results:**

Our model accurately predicts T-cell factor (TCF) activity in mutant cells that vary in APC mutation. Model simulations suggest that both sFRP1 and DKK1 can reduce TCF activity in APC^1638N/1572T^ and Apc^min/min^ mutants, but restoration of normal activity levels is possible only in the former. When applied in combination, synergism between the two inhibitors can reduce their effective doses to one-fourth of the doses required under single inhibitor application. Overall, re-establishment of normal Wnt pathway activity is predicted for every APC mutant in whom TCF activity is increased by up to 11 fold.

**Conclusions:**

Our work suggests that extracellular inhibitors can effectively restore normal Wnt pathway activity in APC-truncated cancer cells, even though these LoF mutations occur downstream of the inhibitory action. The insufficient activity of the truncated APC can be *quantitatively* balanced by the upstream intervention. This new concept of upstream intervention to control the effects of downstream mutations may be considered also for other partial LoF mutations in other signaling pathways.

## Introduction

Mutations in tumor suppressor genes are a hallmark of human cancers. These mutations lead to tumorigenesis by preventing production of proteins which inhibit cell proliferation, or by impairing their normal functionality. Loss of function (LoF) of key tumor suppressor genes, such as BRCA1/2, p53, or Adenomatous polyposis coli (APC), has been intensively investigated in the last decade, and the molecular activity of the products of these genes has been considerably elucidated. A significant investigational effort has been invested in the attempts to develop therapeutic strategies for restoring tumor suppressor activity in LoF mutants, but no method to overcome the tumorigenic effects of mutations has been successfully translated to the clinic, as yet [1–3].

The most frequently mutated tumor suppressor gene in human cancers is APC [4, 5], which is part of the β-catenin destruction complex in the canonical Wnt pathway [6, 7]. The protein β-catenin controls T-cell factor (TCF) activity that regulates the expression of proteins which, in turn, control cell proliferation and differentiation [8, 9]. Truncating mutations in APC, causing LoF of the protein, are found in the vast majority of colon cancer tumors and in many other cancers. In colon cancer, these mutations were characterized both in patients with familial adenomatous polyposis (FAP) and in sporadic colorectal cancers [10, 11]. Most of the somatic APC mutations occur within a small part of the gene–the so-called “mutation cluster region" (MCR), resulting in truncations of about 50% of the APC protein [10]. The resulting truncated protein still retains some activity in the process of β-catenin down-regulation [12–14].

Can the cancerous effects of truncation mutations in APC be prevented? The search for inhibitors of Wnt pathway activity in colon cancer cells bearing APC mutations is guided by the concept that only treatments which affect components of the Wnt pathway *downstream* of APC can be efficient [15, 16]. Several such agents have been developed, some of them reaching the stage of clinical trials. However, none of these agents has been approved for clinical use [2, 15]. In contrast, drugs that affect the Wnt pathway upstream of APC truncation have not been tested for use in cancers harboring APC mutations [16], even though one of these agents, Secreted Frizzled-Related Protein 1 (sFRP1), was found effective in preclinical tumor models [17, 18].

The idea that targets for intervention can be found only downstream of the mutation dominates targeted drug development, in general, and is not unique to the search for inhibitors of Wnt pathway activity [2, 3, 19]. This concept is based on the hypothesis that any intervention upstream of the mutated protein in the pathway will be annulled by the mutated activity downstream. Developing therapeutic agents based on this concept is difficult, for example, because downstream effectors require penetration of the agent into the cell, and sometimes even into the nucleus. Here, we challenge this concept and, alternatively, propose to examine the possibility of reversing the effect of some partial LoF mutations by *upstream* intervention. Our underlying assumption is that when mutations do not entirely eliminate the protein function, but only reduce its activity, upstream intervention may counterweight this reduction by changing the quantitative balance of protein levels in the cell.

Specifically, in this work we study the possibility that treatment by the extracellular inhibitors of the Wnt pathway sFRP1 or Dickhopf1 (Dkk1) could restore normal levels of TCF activity in APC-mutated cells. We do so by extending a mathematical model we have formerly developed for the canonical Wnt pathway in non-mutated cells [20], to describe the aberration caused to the Wnt pathway activity due to different APC mutations. Several mathematical models of the canonical Wnt pathway have dealt with the effects of APC mutations or concentration changes (e.g. [21–23]). These models, like most of the mathematical models of Wnt signaling, were limited to the intracellular components of the pathway, and did not include the effects of extracellular inhibitors (see reviews [24, 25]). This focus is in accordance with the common concept of looking for inhibitors downstream of the mutation.

Our previous work [20], was first in considering a WNT signaling pathway model which also includes the effects of extracellular inhibitors of sFRP1 and Dkk1 binding to membranal receptors [26]. The model was calibrated using *in vitro* experimental data, and its predictive accuracy was demonstrated [20]. Here, we model the effects of sFRP1 and Dkk1 on a range of known and putative APC mutations with different extent of LoF and, therefore, different levels of TCF activity. Our analysis includes two specific murine mutations, *Apc*^*min*^ and *Apc*^*1572T*^, which serve as important experimental models for colorectal cancer. Mouse *Apc*^*min*^, truncated at codon 850 produces a much shorter APC protein than those typical of human colorectal cancer [27]. *Apc*^*1572T*^ harbors truncation mutation close to the MCR, and therefore is translated to APC protein that resembles that in human colorectal cancer. Note that APC^1572T^ is characterized by intermediate level of Wnt/β-catenin signaling activation, higher than wild type (WT) but significantly lower than APC^min^ [28–30]. Based on numerical simulations, we predict that in APC-mutated cells with elevated TCF activity in intermediate levels, extracellular inhibitors can effectively downregulate TCF activity to normal levels.

## Results

### Model validation

**Fig 1:**
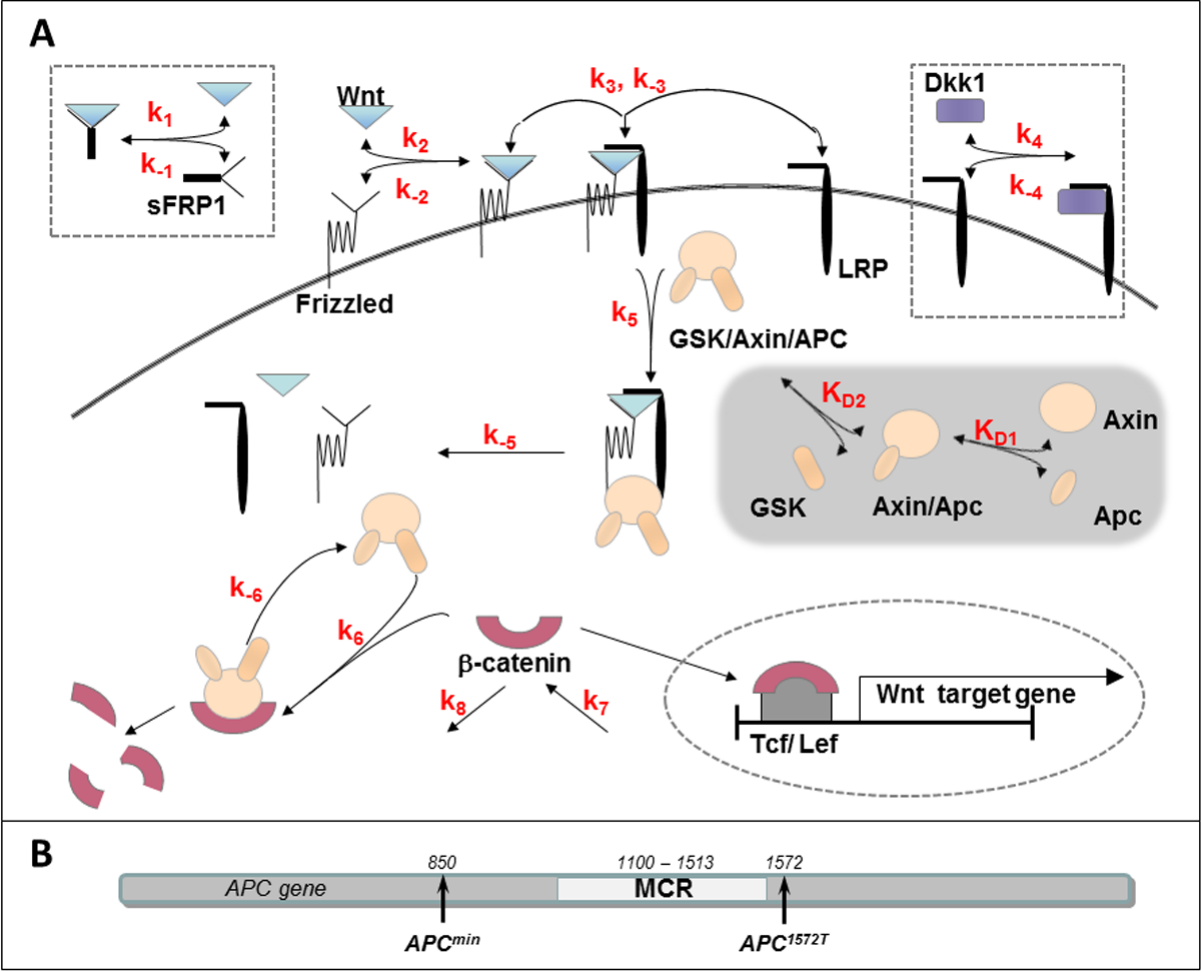
A schematic description of the mathematical model for the Wnt signaling pathway. (A) The central part of the scheme shows the regulation of the β-catenin level in the cell. This process is described by the following cascade of reactions. The Wnt ligand binds to the Frizzled receptor (reaction labeled k_±2_). The resulting receptor–ligand complex may recruit an unoccupied LRP receptor and create a ternary complex consisting of Wnt, Frizzled and LRP (k_±3_). The latter complex transduces the signal inside the cell and interferes with the destruction cycle of β-catenin, by binding a specific destruction complex comprising Axin, APC and GSK3β (k_±5_). This complex regulates the intracellular level of β-catenin; when unbound to the Wnt/Frizzled/LRP ternary complex, the destruction complex binds β-catenin and causes its phosphorylation (k_±6_). Phosphorylated β-catenin dissociates from the destruction complex and is rapidly degraded. The reverse rate constants of these reactions are denoted by a minus in the subscript. Production and degradation of β-catenin, independent of the destruction complex, are also modelled and their rates are labelled k_7_ and k_8_, respectively, and the circled part shows β-catenin function inside the nucleus as TCF activator. The greyed part describes the formation of the destruction complex (GSK/Axin/APC); K_D1_ and K_D2_ are dissociation constants of APC from the Axin/APC dimer, and of the dimer from the destruction complex, respectively. The boxes show reactions between SFRP and Wnt and Dkk1 and LRP receptor, with the rates k_±1_ and k_±4_, respectively. The circled part shows β-catenin function inside the nucleus as TCF activator. (B) A schematic illustration of the APC gene, showing the location of the 1572T mutation, truncated close to the MCR, and of the *min* mutation. These mutations form a truncated APC protein, lacking more functional binding sites as the gene is truncated closer to the translation starting site. Note that all other mutations referred to in this work are not known to form a truncated APC protein, but rather attenuate the expression of the full length APC protein.

In order to study the response of APC-mutated cells to extracellular inhibitors, a previously validated model for the canonical Wnt pathway [20] has been extended to include the effects of β-catenin on TCF activity level, as well as the formation of normal and aberrant β-catenin destruction complexes (Fig 1A; Materials and Methods). The new model describes the destruction complex in cells containing the APC mutation, which differs from the destruction complex in WT cells in its concentration and in its potency to bind β-catenin and cause its degradation. Mutation-specific parameters for the APC protein were implemented in the new model, enabling us to model specifically truncated proteins APC^1572T^ and APC^min^ (see Fig 1B), as well as various hypothetical mutations.

**Fig 2:**
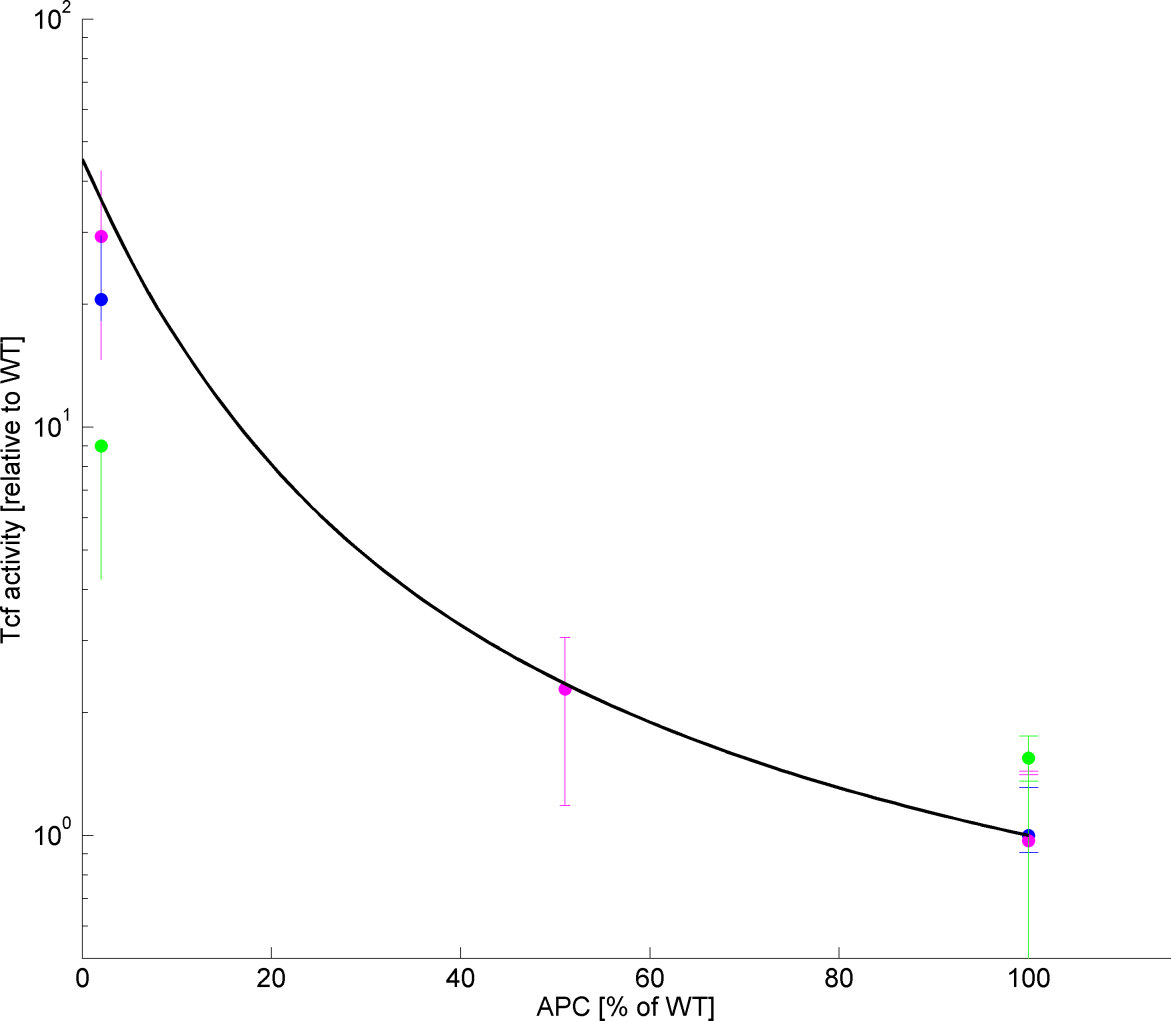
Model validation by comparison of the predicted effects of attenuated full-length APC on TCF activity levels, to experimental data. Simulation results (black line) for cells with reduced APC expression are presented in comparison with the experimental results for cells with the mutations 1638N/1638N, 1638N/1638T and 1638T/1638T, which express APC at 2%, 51% and 100% of the WT level, respectively. The observed average TCF activity levels are taken from [34] (magenta circles), [29] (blue circles) and [33] (green circles); error bars are reproduced from the original publications. The discrepancies between the experimental results from different sources may be due to differences in the time of exposure to Wnt. The simulated and experimental values of TCF activity level are detailed in S1 Table for each specific mutation.

The new model was first calibrated for cells with non-mutated APC (see Materials and methods and S1 Text; parameters shown in Table 1; note that β-catenin concentrations are translated to the experimental measurement units in [31] using the multiplicative scale parameter ***λ*,** as in [20]). Thereafter, it was simulated for different mutations, which do not cause truncation of the protein, but rather attenuate its expression. These mutations were represented in the model as a reduction in the concentration of total APC (*P*_*T*_), all other parameters maintaining WT values (See Table 2). The level of TCF activity was calculated as a function of the concentration of fully active APC in the cell. As Wnt level in the experimental setting is unknown, it was calibrated using experimental data for neoR/neoR and neoF/neoF mutants, which reduce the expression of full-length APC protein to 20% and 10% of the level in WT cells [32] (See Materials and Methods). Model simulations were compared to experimental results for cells with one or two alleles bearing the 1638N mutation, which reduces the expression of full-length APC protein to 2% of the level in WT cells [29, 33, 34]. As can be seen in Fig 2, the lower the concentration of fully active APC, the higher is the level of TCF activity. Importantly, Fig 2 demonstrates the high precision of the model-calculated values of TCF activity, as compared to experimental results from different sources. These results validate the quantitative accuracy of our model in predicting TCF activity levels, as affected by reduced concentrations of fully active APC. The observed discrepancy between the experimental values of TCF activity levels from different sources, may result from differences in the experimental assays, e.g., in the time elapsing from the transfection to the TCF activity assay [29, 33, 34]. Since our simulations suggest a much slower conversion to the steady state in mutant cells (lasting more than 48 hours) than in WT, especially at high Wnt concentrations (data not shown), it is plausible that steady state was not reached in some of the experiments. Due to insufficient information on experimental times (e.g. [33]), in all our simulations we assumed that steady state was reached.

**Table 1:** The parameters of the model and their evaluated values for WT APC.

parameter	value (and reference)		Units	Biological meaning
***k***_*1*_	4.33·10^4^ [35]		*M*^-1^·*s*^-1^	sFRP1-Wnt binding rate
***k***_*-1*_	4.86·10^−4^ [35]		*s*^-1^	sFRP1-Wnt dissociation rate
***k***_*2*_	7.9·10^4^ [35]		*M*^-1^·*s*^-1^	Wnt-Frizzled binding rate
***k***_*-2*_	4.7·10^−4^ [35]		*s*^-1^	Wnt-Frizzled dissociation rate
***k***_*3*_	1.3·10^7^ (Fitted to [31])		*M*^-1^·*s*^-1^	Frizzled-LRP binding rate
***k***_*-3*_	0.1[36]		*s*^-1^	Frizzled-LRP dissociation rate
***k***_*4*_	1.03·10^6^ [37]		*M*^-1^·*s*^-1^	Dkk1-LRP binding rate
***k***_*-4*_	5.05·10^−4^ [37]		*s*^-1^	Dkk1-LRP dissociation rate
***k***_*5*_	4.84·10^5^ (Fitted to [31])		*M*^-1^·*s*^-1^	Ternary complex-destruction complex binding rate
***k***_*-5*_	10^−4^ [38]		*s*^-1^	Ternary complex-destruction complex dissociation rate
***k***_*6*_	2.72·10^5^ (Fitted to[31])		*M*^-1^·*s*^-1^	Destruction complex-β-catenin binding rate
***k***_*-6*_	1.25 (Fitted to[31])		*s*^-1^	Destruction complex-β-catenin dissociation rate
***k***_*7*_	1.39·10^−9^ (Fitted to[31])		*M*·*s*^-1^	β-catenin production rate
***k***_*8*_	4.2·10^−6^ [39]		*s*^-1^	β-catenin degradation rate
***F***_*T*_	1.01·10^4^ (Fitted to[31])		*receptor/cell*	Total number of Frizzled
***L***_*T*_	4·10^3^ [37]		*receptor/cell*	Total number of LRP
***C***_*T*_	8.05·10^−9^ (calculated from [40])		*M*	Total destruction complex
***P***_*T*_	3.4·10^−8^ [40]		*M*	Total APC
***A***_*T*_	3·10^−8^ [40]		*M*	Total Axin
***G***_*T*_	2.1·10^−8^ [40]		*M*	Total GSK
***K***_*D1*_	50 [39]		*M*	Dissociation constant of APC from Axin/APC
***K***_*D2*_	10 [39]		*M*	Dissociation constant of Gsk from destruction complex
***V***_*cell*_	3.5·10^−13^ [41]		*l*	Cell volume
**Experiment-specific parameters**	**Hannoush experiments (for calibration)**	**All other simulations**		
***W***_*T*_	0 to 10^−8^ [31]	5·10^−9^ (fitted to [32])	*M*	Total Wnt
***S***_*T*_	0	0 to 5000 nM	*M*	Total sFRP1
***D***_*T*_	0	0 to 500 nM	*receptor/cell*	Total Dkk1
***V***_*exp*_	2·10^−5^ [31]	3·10^−3^ [42]	*l*	Volume of experimental well
***N***_*cell*_	5·10^3^ [31]	10^6^ [42]	*cell*	Cell number
***a***	—	1.51 (fitted to [32])	*unitless*	Rank of β-catenin–TCF power-law relation
***λ***	2.2·10^4^ (Fitted to [31])	—	*measurement units/M*	Translation of β-catenin concentration to experimental measurement units in [31]

Parameter values were estimated directly from literature, or by curve fitting to experimental data. Experiment-specific parameters are shown separately for each of the simulated setups.

**Table 2:** Different APC mutations and their effect on values of model parameters.

Mutation	Description of effect of one allele mutated	*P*_*T*_ / *P*_*T WT*_	*k*_*6*_ / *k*_*6 WT*_	*K*_*D1*_ / *K*_*D1 WT*_
WT	–	1	1	1
neoR	Reduces expression of full-length APC protein by the mutated allele to 10%	0.1	1	1
neoF	Reduces expression of full-length APC protein by the mutated allele to 5%	0.05	1	1
1638T	Negligible effect–equivalent to WT	1	1	1
1638N	Reduces expression of full-length APC protein from the mutated allele to 1%	0.01	1	1
1572T	Produces a truncated APC protein, which reduces its affinity to Axin (increases *K*_*D1*_), or affinity of the destruction complex to β-catenin (decreases *k*_*6*_), or both.	1	0.05 *≤k*_*6*_/*k*_*6WT*_ *≤* 1, obeying Eq (1)	0.05 ≤ *K*_*D1WT*_/*K*_*D1*_ ≤ 1, obeying Eq (1)
min	Produces a truncated APC protein, which reduces its affinity to Axin (increases *K*_*D1*_), or affinity of the destruction complex to β-catenin (decreases *k*_*6*_), or both.	1	0.05 *≤ k*_*6*_/*k*_*6WT*_ *≤* 1, obeying Eq (2)	0.05 ≤ *K*_*D1WT*_/*K*_*D1*_ ≤ 1, obeying Eq (2)

Model parameter values *P*_*T*_, *k*_*6*_ and *K*_*D1*_ for each APC mutation are shown relative to *P*_*T WT*_, *k*_*6WT*_ and *K*_*D1WT*_, which are the values of the these parameters in WT APC (see Table 1). The rest of parameters are assumed to be unaffected by the mutations. For heterozygous cells, two different parameter values are used for the two types of APC proteins coexisting in the cell (See Materials and Methods). In such a case, one of them is denoted with a prime, e.g., *P*_*T*_ ≠ *P*_*T*_'. For a homozygous cell only one parameter is used, without a prime.

### Calculation of binding parameters in truncated APC mutants

The impact of truncation mutations on an APC allele is reflected in the model either by decreasing concentration of the destruction complex APC/Axin/GSK, modeled by increasing *K*_*D1*_, or by decreasing affinity of this complex to β-catenin, modeled by decreasing *k*_*6*_, or by both effects (see Materials and Methods and Table 2). We used experimentally measured TCF activity levels in embryonic stem (ES) cells with APC^1638N/1572T^ [34], or APC^1572T/1572T^ [33], to calculate the aberrant parameters of the APC^1572T^ protein, truncated close to the MCR (see Fig 1B), namely, its affinity to Axin (*K*_*D1*_) and the affinity of the destruction complex to β-catenin (*k*_*6*_). The parameters were calibrated by simulating TCF activity levels in mouse ES cells which carry these mutations, and comparing to the experimental data (see Materials and Methods). Our results show that multiple different pairs of values for the parameters *K*_*D1*_ and *k*_*6*_ allow the model to be equally successful in retrieving the experimental results of mutant cells, bearing the APC^1572T^ protein. The constraint on the values of these parameter pairs is that they are positive and obey Eq (1):

$$\left(\frac{k_{6}}{k_{6WT}}\right)=0.27\left(\frac{K_{D1}}{K_{D1WT}}\right)+0.26,$$ (1)

where *k*_*6WT*_ and *K*_*D1WT*_ are the values of the parameters *k*_*6*_ and *K*_*D1*_ in WT APC (see Tables 1 and 2). Eq (1) determines the range within which the values of *k*_*6*_ and *K*_*D1*_ may vary for cells bearing this mutation, and our simulations show that variation within this range does not alter model predictions of TCF activity (not shown). Below, we simulated the Wnt pathway activity in inhibited APC^1572T^ cells under the range of values of *K*_*D1*_ and *k*_*6*_ that obeys Eq (1).

In the same way, we used experimental measurement of TCF activity level in APC^min/min^ cells [29] to evaluate *K*_*D1*_ and *k*_*6*_ in the truncated APC^min^ protein. As in the former case, here too, simulations reproduce experimental data with infinitely many pairs of positive values of *K*_*D1*_ and *k*_*6*_, which can vary according to

$$\left(\frac{k_{6}}{k_{6WT}}\right)=0.004\left(\frac{K_{D1}}{K_{D1WT}}\right)+0.007.$$ (2)

These results serve for simulating the effects of inhibitors on the studied mutant cells.

### Effects of sFRP1 and Dkk1 in cells with specific APC mutations

**Fig 3:**
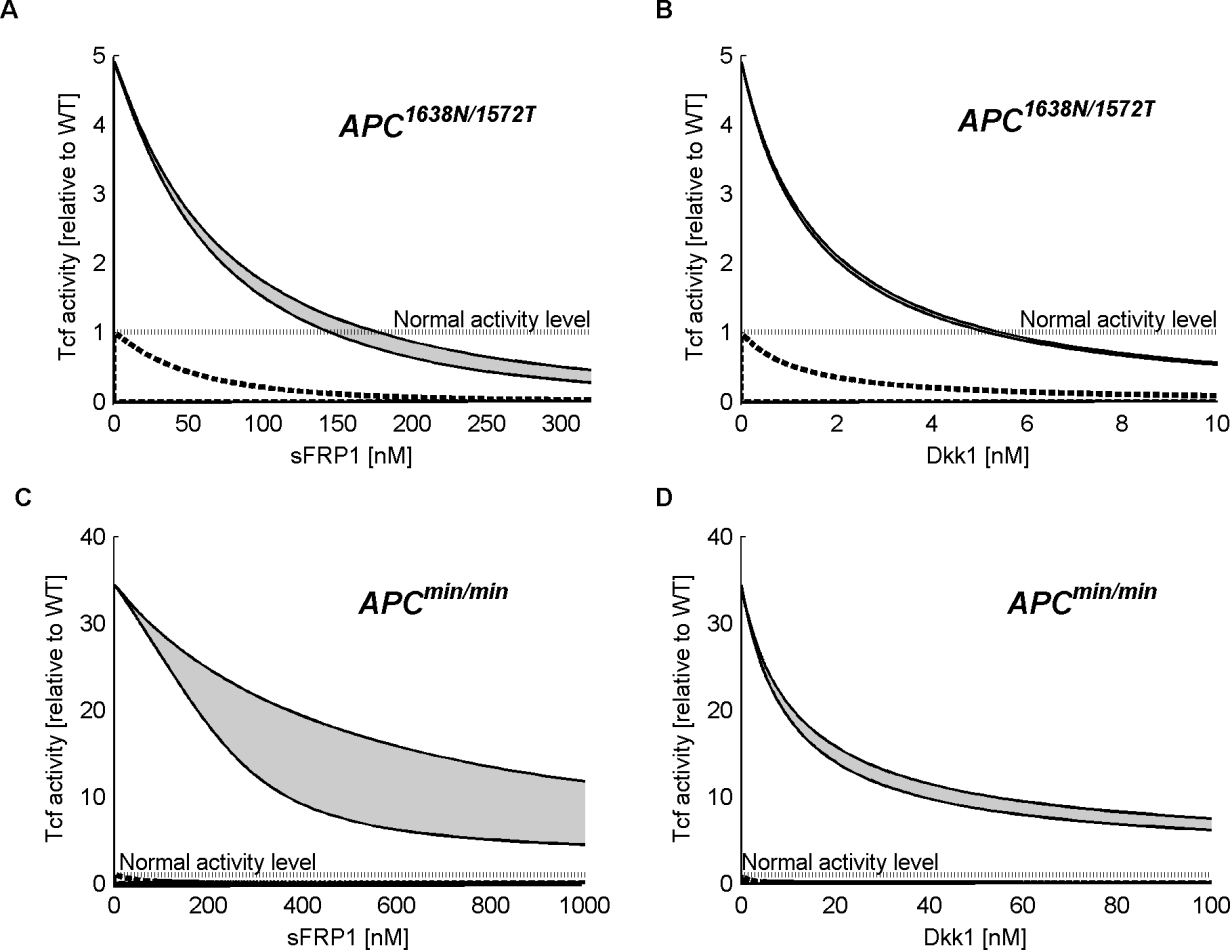
Model–predicted effects of sFRP1 and Dkk1 on cells with APC truncation mutations. Model predictions of TCF activity under inhibition by sFRP1 or Dkk1 in differently mutated cells are shown; the grey area marks the level of inhibition by each inhibitor. In APC^min/min^ mutant cells, neither of the inhibitors restores normal TCF activity level (Fig 3A and 3B). Restoration of normal TCF activity level (i.e., equal to that of WT) is predicted to be achievable in APC^1638N/1572T^ mutant cells (grey area), by addition of either sFRP1 (~160nM) or Dkk1 (~5nM) (Fig 3A and 3B, respectively). For comparison, simulations of the effects of inhibitors on WT APC under the same conditions are shown in all four panels (dashed lines). TCF activity levels are relative to WT.

To simulate the effects of sFRP1 or Dkk1 on TCF activity in APC^1638N/1572T^ and APC^min/min^ mutant cells, we evaluated the binding parameters of the proteins translated from *Apc*^*1572T*^ and *Apc*^*min*^ alleles, using Eqs (1) and (2). Predicted dose-responses are shown in Fig 3, both for APC^1638N/1572T^ cells with sFRP1 (Fig 3A) and Dkk1 (Fig 3B), and for APC^min/min^ mutant cells with sFRP1 (Fig 3C) and Dkk1 (Fig 3D). In all these cases, simulations with different parameter values within the range obeying (1) or (2), result in a range of predictions for TCF activity. Dose-response curves of both inhibitors show a similar pattern in WT APC and in mutated cells: Dkk1 is more efficient than sFRP1 in reducing TCF activity level (in agreement with the observed experimental data; see Figs 5 and 7 in [20]), the inhibitory effect being asymptotically limited. That is to say that the effect of the increase in dose is smaller for higher applied doses. Applying a sufficiently large dose of either inhibitor to cells bearing the mutation APC^1638N/1572T^, reduces TCF activity to its level in untreated WT cells. In contrast, in APC^min/min^ mutant cells, even high concentration of sFRP1 or Dkk1 cannot restore the levels of TCF activity in WT cells. Model predictions of the doses of sFRP1 and Dkk1 that are required for restoring WT TCF activity (*effective doses*) in APC^1638N/1572T^ cells, are reported in Table 3 (5^th^ row) with the related parameter-determined range.

**Table 3:** Effective doses of sFRP1 and Dkk1 for different mutants as predicted by the model.

Mutation	TCF activity level (relative to WT)	Effective sFRP1 dose (nM)	Effective Dkk1 dose (nM)	effective sFRP1–Dkk1 synergistic combination (nM sFRP1, nM Dkk1)
WT (+/+)	1.0	0	0	(0,0)
1572T/1572T	2.2	54–55	1.5–1.6	(19,0.6)—(22,0.5)
1638N/1638T	2.4	63	1.7	(25,0.5)
min/+	2.4–2.7	66–69	1.8–2.1	(22,0.7)—(26,0.6)
1638N/1572T	4.9	148–178	5.2–5.5	(39,1.5)—(45,1.4)
neoR/neoR	8.1	372	12.0	(76,2.2)
neoF/neoF	16.4	>3000	110	(242,8)
min/min	34.5	*Inf*	*inf*	*inf*
1638N/1638N	36	*Inf*	*inf*	*inf*

Shown in the table are values of TCF levels in absence of inhibitors, under specific experimental conditions. Effective inhibitor doses are those required to restore TCF activity levels in WT cells with no inhibitor added. The last column shows the maximally synergistic combination of sFRP1 and Dkk1, as defined in [20].

Simulations of the combined effects of sFRP1 and Dkk1 on APC^1638N/1572T^ mutant cells suggest synergism between the two inhibitors, as was found in WT cells (cf. Fig 8 in [20]). The synergistic effect appears across all the parameter range (S1 Fig). Shown in Table 3 are the maximally synergistic doses of sFRP1 and Dkk1 which, in combination, can restore TCF activity to that of the WT. The effective doses of sFRP1 and Dkk1 in this combination are only a quarter of their effective doses when each inhibitor is applied alone.

By substituting into the model the above calculated values of *K*_*D1*_ and *k*_*6*_, characterizing specific mutations in APC, we could predict TCF activity levels and the effects of inhibitors in cells carrying these mutations in any combination of alleles. For example, we used parameter values of *APC*^*min*^, *APC*^*1572T*^, as determined by Eqs (1) and (2) above, to simulate TCF activity levels in APC^min/+^ and APC^1572T/1572T^ mutant cells. For each of these mutants, addition of Dkk1 or sFRP1 is predicted to reduce TCF activity to WT level (effective doses shown in Table 3). In addition, we simulated the inhibitors' effects in the cell lines APC^neoR/neoR^ and APC^neoF/neoF^. As mentioned above, these mutations reduce the expression of full-length APC protein but do not cause its trunctation [32]. Hence they were modeled as a reduction in the concentration of total APC (*P*_*T*_), parameters *K*_*D1*_ and *k*_*6*_ maintaining WT values. In general, when TCF activity in the untreated mutated cell increases, larger doses of the inhibitors are required for buffering the harmful effects of mutations on the process of β-catenin degradation (Table 3). However, above a certain threshold of mutational effects, normal levels cannot be fully restored by any concentration of the inhibitors, although TCF activity levels are somewhat reduced. These findings are supported by the results of model sensitivity analysis (see S2 Text).

### Effects of sFRP1 and Dkk1 on a range of hypothetical APC mutations

To represent various hypothetical truncation mutations, the values of *k*_*6*_ and *K*_*D1*_ were set within a wide range for each of the two types of the APC protein in the cell. Generally, the effect of the mutation (expressed as higher TCF activity level) increases with increasing dissociation rate of APC from Axin (*K*_*D1*_), or with decreasing affinity of destruction complex to β-catenin (*k*_*6*_).

We simulated three possible mutational scenarios. The first scenario involves heterozygous mutations, with one allele truncated (different truncation levels represented by a range of possible values of *K*_*D1*_ and *k*_*6*_), and the other is practically inactive (parameter values set equal to those of APC^1638N^, which represents a null allele [29]). This could represent human cancerous cells, in which one allele is genetically truncated, and the second allele is deleted as a result of loss of heterozygosity (LOH). The second scenario implicates homozygous truncation mutations, which in human cancer cells can result from copy-neutral LOH, caused by uniparental disomy [43–45]. The third scenario refers to mutations that reduce the expression of full-length APC protein (represented by different values of *P*_*T*_) but do not cause its truncation, like those experimentally studied in [32]. We simulated each hypothetical scenario with parameter values within a wide range, and mapped each mutated cell onto the scale of its cellular TCF activity level, in the absence of inhibitors. For each mutated cell, we also evaluated the effects of sFRP1 or Dkk1 in different doses, and determined the effective doses required to reduce TCF activity to the WT level. This exercise includes many putative mutants, each one having specific values for *P*_*T*_, *K*_*D1*_ and *k*_*6*_ for each of the two alleles.

**Fig 4:**
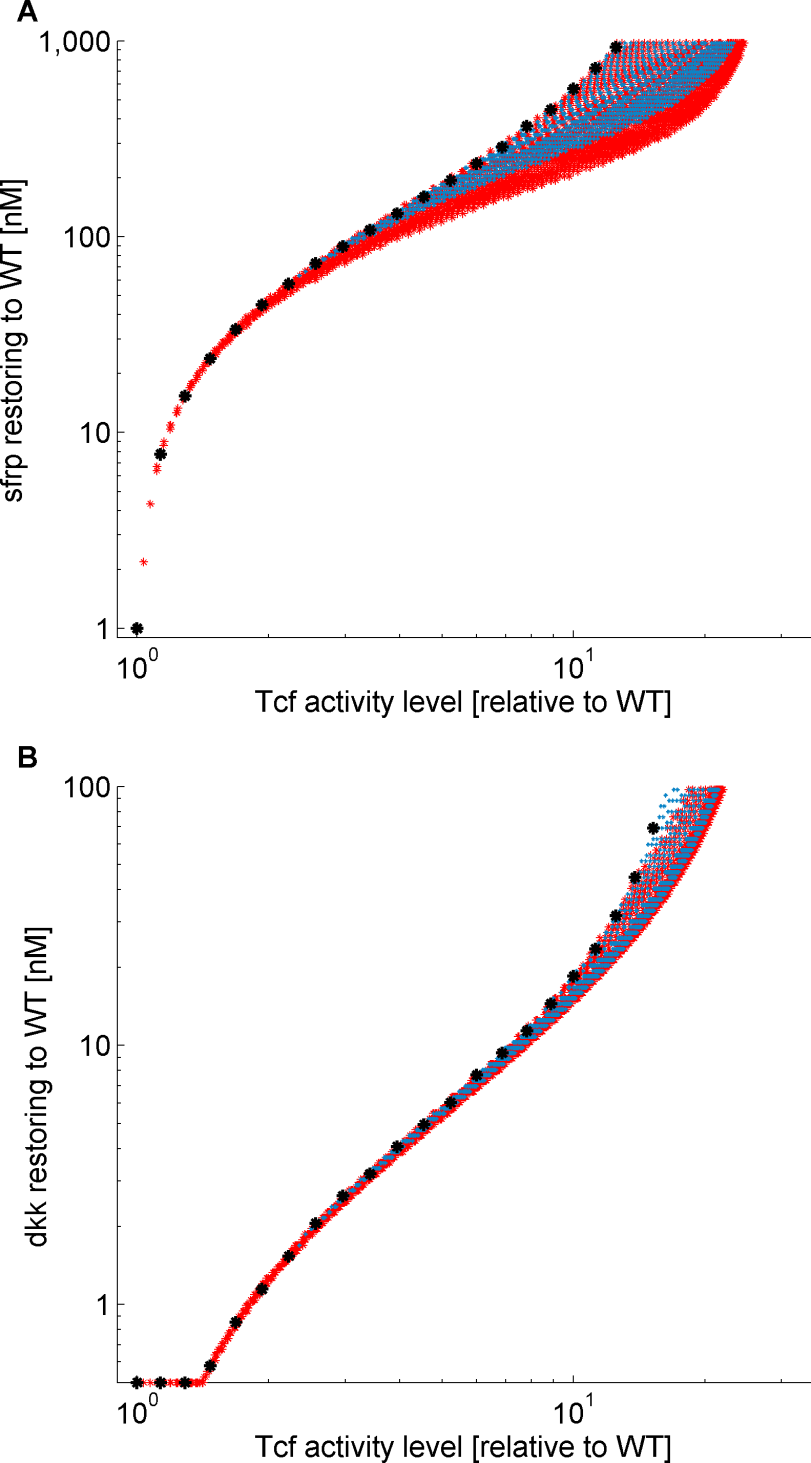
**Effective doses of sFRP1 (Fig 4A) and Dkk1 (Fig 4B), required to restore normal TCF activity level, predicted for hypothetical mutations of different severity.** Shown are simulation results for cells bearing different mutations: heterozygous mutations, with one APC allele truncated and the other practically inactive (blue dots), homozygous truncation mutations (red dots), and mutations with reduced amount of normally-functioning APC (black stars). The severity of mutation is expressed by the increase in TCF activity level. The range of inhibitor doses which are effective for each TCF activity level is due to the fact that the same level of increase in TCF activity can result from different truncation mutations (having different parameter values within the biologically plausible range).

In Fig 4, we mapped the model-evaluated effective doses of sFRP1 and Dkk1 (vertical axis) as evaluated for the different mutated cells, on the cellular TCF activity level in the untreated mutated cell, signifying the severity of the putative mutation (horizontal axis). For both heterozygous and homozygous mutations (blue and red dots, respectively), there exists a range of possible effective doses of the inhibitors for any mutation with a certain TCF activity level, as already noted when specific mutations were studied. This range is very narrow for minor truncation mutants, but widens when the mutation is more harmful, i.e. when TCF activity is considerably elevated, in the absence of inhibitors. Generally, the effective doses of inhibitors are correlated to TCF level in the mutated cells, and can be evaluated based on this level, measured experimentally; the same doses of inhibitors will have the same efficacy in all mutated cells having the same TCF activity level when untreated.

The effective doses for the third kind of mutants, with reduced expression of full-length APC protein, can be placed on one edge of the effective doses range for cells with truncation mutations (black stars in Fig 4). This is because such mutations can be represented by variation of only one parameter, *P*_*T*_, which has the same effect as changing *K*_*D1*_ (equivalent to the special case of truncation mutation which only affects *K*_*D1*_ and not *k*_*6*_).

Taken together, these results suggest that restoration to normal TCF activity level is possible in APC mutants in which TCF activity level is increased by up to a given threshold (equaling at least 11 fold) relative to the WT, and this does not depend on the specific molecular mechanism underlying the reduction of APC functioning. This threshold is still below the TCF activity level in APC^min/min^ mutants, so that the damage caused by this mutation is not expected to be buffered by extracellular inhibitors. In contrast, the threshold is sufficiently high to include the activity levels in cells with APC-mutated close to the MCR region, typical to human oncogenic mutations, e.g. APC^1638N/1572T^, and cells bearing mutations of larger TCF activity, such as APC^neoR/neoR^ and APC^neoF/neoF^ (see Table 3).

## Discussion

**Fig 5:**
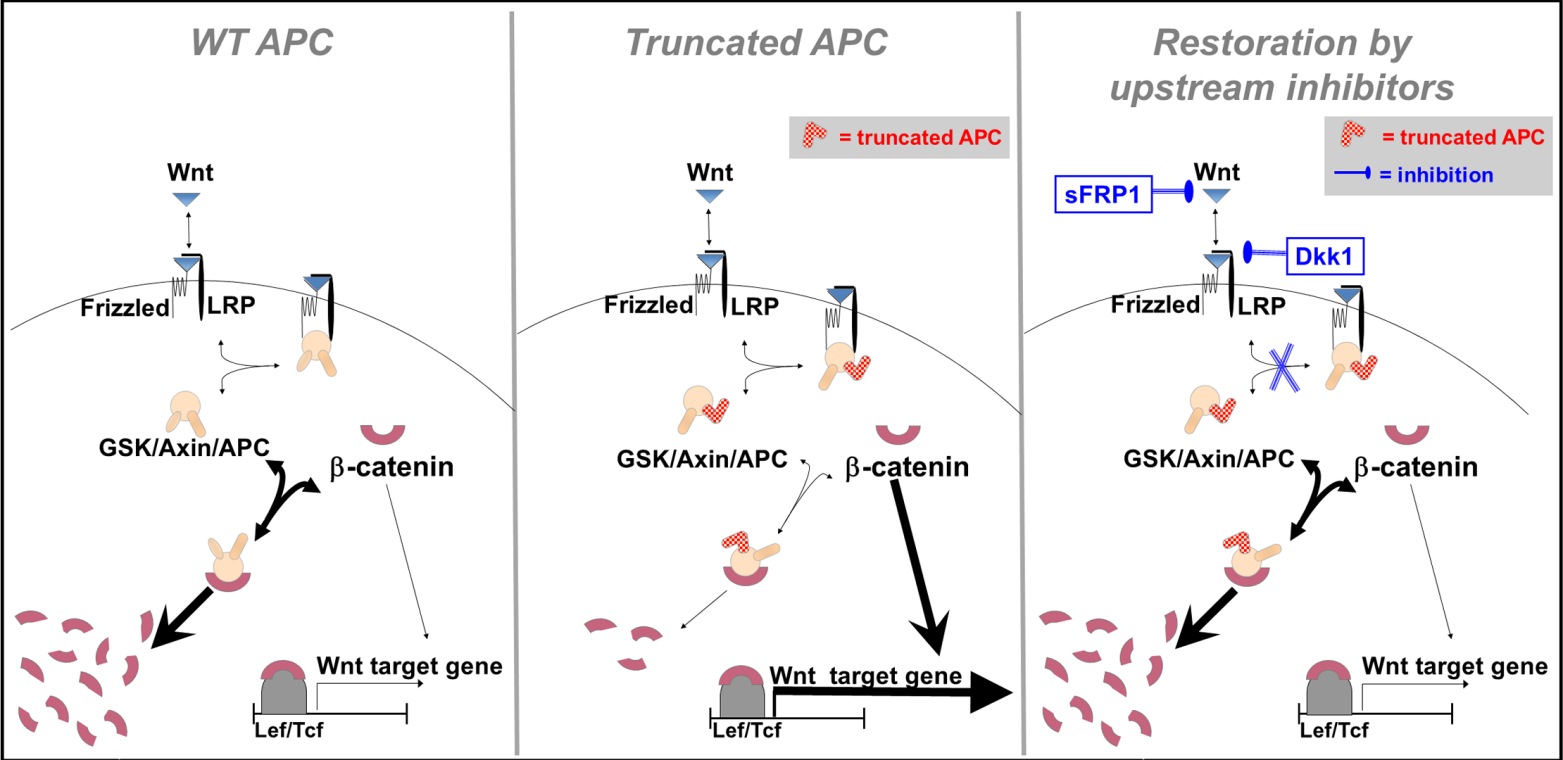
A simplified scheme of the Wnt pathway, figuring restoration of normal activity in APC-mutated cells by extracellular inhibitors. In normal cells (left), β-catenin is downregulated by the destruction complex GSK/Axin/APC. When APC is truncated (middle), β-catenin is accumulated in the cell and Wnt target genes are excessively expressed, leading to uncontrolled proliferation. Extracellular inhibitors of the Wnt pathway may reestablish the quantitative protein balance and restore normal Wnt activity levels (right), by reducing the number of Wnt receptors which bind the destruction complex, leaving more of the destruction complex free to degrade β-catenin.

Our results suggest that extracellular inhibitors can effectively restore normal Wnt activity in APC-mutated cancer cells, even though these mutations occur downstream of the inhibitory action. This is because, in the pertinent APC truncation mutations, the loss of function is only partial, and the mutated APC retains some activity in the process of β-catenin downregulation [13, 14]. For this reason the mutational effect can still be effectively balanced by extracellular inhibitors. Indeed, recent experiments showed that truncated APC retains partial functionality in binding to Axin, GSK and β-catenin, and that even in the presence of APC mutations, the signaling is still dependent on Wnt ligands [46]. Hence, although the amount of the destruction complex, or its affinity to β-catenin, is reduced in the mutated cells, β-catenin accumulation can be compensated by increasing the amount of *free* destruction complex. Complying with the experimental results, model sensitivity analysis shows that of all the model parameters, the destruction complex concentration exerts the largest effect on β-catenin accumulation (see S2 Text). This reduction of destruction complex concentration can be achieved through addition of inhibitors that reduce the number of Wnt-occupied receptors (ternary complex), which bind the destruction complex, and thus more of it is left free to phosphorylate β-catenin, as illustrated schematically in Fig 5. We predict that in given settings, depending on the level of APC loss of function, it is possible to effectively restore normal levels of pathway activity in the mutated cells.

The suggestion that extracellular inhibitors can be efficacious in APC-mutated cells stands in contrast to the current view, according to which such cells can only be controlled by treatments that affect downstream components of the Wnt pathway, e.g. prevention of transcriptional activity of TCF, or disruption of β-catenin/TCF binding by small molecules [2, 14–17]. Intervention downstream to APC is challenging, as it requires penetration of the inhibitor through the cell membrane, and sometimes also through the membrane of the nucleus. In addition, any direct intervention in β-catenin activity, not specifically through Wnt pathway, is likely to be cytotoxic, because of the multiple pivotal roles of β-catenin in other contexts, where it is regulated through other pathways [2, 47]. Revoking the limitation to search only downstream of Apc enables researchers to expand the range of admissible therapeutic targets, potentially allowing development of new extracellular inhibitors.

Development of extracellular inhibitors, such as sFRPs and Dkk1, as targeted therapy for tumorigenic APC-mutated cells, looks promising, because their delivery is more doable since their activity does not require laborious penetration through membranes. Moreover, these treatments are not expected to cause significant toxicity, since the Wnt pathway is usually active only in stem cells, and in adults it is involved mainly in tissue repair [48]. In addition, it was already shown that extracellular inhibitors can be effective as anti-cancer agents: inhibition of the Wnt–induced signaling pathway by restoring expression of sFRPs attenuates tumorigenicity in various cancer cells [18]. Furthermore, it was shown that restoration of sFRP or treatment with a WNT1 antibody decreases β-catenin stabilization and attenuates tumorigenic behavior, even when downstream components of the canonical pathway are mutated [13, 14]. Taken together, these potential benefits lend support to our suggestion to examine the use of these inhibitors as anti-cancer agents also in tumors comprising mutated APC.

We used our mathematical model to predict the effect of extracellular inhibitors on the pathway activity in cells harboring different APC mutations. As model inhibitors, we used sFRP1 and Dkk1 and as model cells, we used known cell lines that mimic the level of LoF of APC in human cancer cells, both with truncated APC (APC^1572T^ [34]) and with attenuated levels of full-length APC (APC^neoR^, APC^neoF^[32]) (see Table 3). We also simulated various hypothetical cells, homozygous or hemizygous (i.e., containing only one copy of the gene,) in APC truncation mutations. We examined mutations that affect both the concentration of the destruction complex and its efficiency in phosphorylation of β-catenin at different intensities (see Fig 4). Our results suggest that sFRP1 or Dkk1 can restore normal Wnt pathway activity in cells in which the superfluous TCF activity does not exceed a threshold of 11 fold increase above the WT level (Fig 4). Included below this threshold of TCF activity are APC^1638N/1572T^ cells, mutated close to the MCR region of the APC gene, which characterizes mutations in many cancer cells [10, 49, 50]. We expect that restoration of normal Wnt pathway activity by the studied inhibitors is also possible in APC-mutated human cancer. This is based on the TCF activity measured in cells carrying relevant APC mutations, being less than 11 fold that in WT cells [32, 34, 51]. This is in line with the ‘just right’ theory that extensive overexpression of β-catenin, which causes strong TCF downstream signals, is unfavorable for tumor formation, because it induces cell apoptosis [12].

In [46] it was shown that reduction of the expression of full length APC increases cell sensitivity to recombinant Wnt, i.e., to upstream signals. Our results suggest that cells with truncated APC are also sensitive to upstream signals: the same concentration of an inhibitor decreases TCF activity of mutated cells more than in WT cells (see Fig 3). However, according to our model predictions, this effect by itself cannot guarantee the achievement of normal TCF levels, since it is limited. In some cases, the maximally possible reduction in TCF activity may still be insufficient to reverse to normal activity.

Our results suggest that the effective dose of the applied inhibitor, required to restore normal TCF activity in mutant cells, should increase with increasing TCF activity level in the cell, i.e. with severity of the LoF of APC. However, as a result of the synergism between Dkk1 and sFRP1, found for all simulated APC mutants, the use of these inhibitors in combination may significantly lower the total effective doses of the applied inhibitors.

Our mathematical model can serve for evaluating the effective doses of the inhibitor(s) in cells carrying any specific APC mutation, based on a single measurement of TCF activity in untreated mutated cells. Even though different mutations induce different effects on the pathway, e.g., decreasing concentration of the destruction complex, decreasing its affinity to β-catenin, decreasing production of APC etc., we found that the inhibitory effect can still be predicted based solely on TCF level in the mutated cells. However, the existence of a potential range of mutations yielding the same TCF activity levels (see Fig 4), introduces some uncertainty in the predictions of the effective inhibitor doses. Yet, this uncertainty is negligible for the mutations that are relevant in colon cancer, as they have intermediate levels of TCF activity. More information on the molecular mechanisms underlying β-catenin regulation is needed for decreasing the uncertainty in mutants with higher TCF activity. At any rate, even when information on the precise downstream activity of specific mutation is lacking, we can predict with acceptable accuracy the inhibitory dose-effect for the relevant mutated cells.

Our theoretical predictions that sufficiently large concentrations of extracellular inhibitors restore normal level of the pathway activity should be tested experimentally in cell lines harboring mutations close to the MCR. Our results suggest that in APC-truncated cells with significantly shorter APC gene, such as APC^min^, restoration to normal levels is not feasible: in these mutants, the reduction of APC functioning is too large to be compensated through extracellular inhibition of Wnt activity. Experimental validation of the results is also important in order to examine our model, which assumes that regulators of the Wnt pathway have a dominant influence on TCF activity, neglecting other pathways that regulate β-catenin (e.g., NFκB and P53), crosstalk between canonical and non-canonical Wnt pathways, or involvement of β-catenin in cell-to-cell interactions [47, 52]. Extending our model to include cellular characteristics, enabling prediction of the effects of TCF activity on cell fate, proliferation or apoptosis, may provide new insights on how extracellular inhibitors affect mutated cells. For example it can examine the "just right" assumption for the selection of APC genotypes that retain some activity of β-catenin signaling [12].

In summary, our findings suggest that treating APC-mutated cells with upstream Wnt inhibitors is a valid option. This increases the scope of potential inhibitors for colon cancer, hopefully resulting in improved treatment efficacy, due to the relative wide availability of extracellular targets, as compared to the availability of targeted therapy directed to downstream locations in the pathway. Using our mathematical model, one can estimate the quantitative level of the inhibitory effect that is sufficient for treatment.

The concept of upstream intervention to control the effects of downstream mutations may be considered also for partial loss of function in other proteins. We hypothesize that this is possible when mutations do not entirely eliminate the protein function, but rather reduce its activity. In such situations, upstream intervention may reestablish the quantitative balance of protein levels in the cell by better use of the proteins that are still functioning. More generally, the underlying rationale of our findings is that the normal cellular activity depends on a quantitative balance of pathway proteins, which may be perturbed by mutations. We propose that any intervention in a mutated pathway, restoring this quantitative balance, is worth considering as a drug candidate, even if it acts upstream of the mutation. This conceptual change would enable consideration of a larger scope of cancer treatment options.

## Materials and methods

### The mathematical model

Our mathematical model for WNT pathway activity in APC mutants, taking the form of a system of ordinary differential equations (ODEs), is schematically described in Fig 1A. This model is an extension of a previous model, describing the regulation of β-catenin levels in wild type cells [20]. In both models, we assumed that the intracellular level of β-catenin is regulated by a specific destruction complex comprising Axin, APC and GSK3β, which binds β-catenin and causes its phosphorylation and degradation [53]. The regulation of β-catenin destruction is carried out in the model via a ternary complex of Wnt with its receptors: Frizzled and LRP [54–56], that binds the destruction complex through the scaffold protein Axin and prevents it from binding β-catenin and inducing its degradation [57, 58].

The current model is new in taking account of the assembly of the destruction complex, which regulates the intracellular level of β-catenin as a function of APC level and structure (see Fig 1A, greyed part). We assumed that the destruction complex is at rapid equilibrium with all its components, and thus its formation is a two-stage reversible process of APC binding to Axin, followed by the binding of the intermediate dimer to GSK3β [39]. In order to model cells with heterozygous mutations, we assumed that two types of APC protein may coexist in the system at varying concentrations. These compete for Axin and GSK to create two types of destruction complex, which differ in their potency to bind β-catenin and cause its degradation.

We also assume that the pathway can be downregulated by sFRP1, which competes with Frizzled on Wnt binding, and Dkk1, which binds to LRP and abolishes formation of the ternary complex [37, 59, 60].

The equation system (3–18) describes Wnt pathway activity in a cell, in which one or two of the APC alleles may be mutated. In the setting of heterozygosity, two different types of destruction complex may coexist in the system (denoted C, C'; see also the explanation for Eqs (19–26) below). In such a case there are also two, possibly different, parameters for affinity of destruction complex to β-catenin (denoted *k*_*6*_, *k*_*6*_*'*). All other model parameters are assumed to be unaffected by APC mutations. The specific case of homozygous cell is represented by setting C_T_' = C' = C_L_' = C_B_' = 0, which effectively cancels all boxed terms and equations. Parameter values for the case of WT APC can be found in Table 1.

$${\dot{S}}_{W}=k_{1}SW-k_{-1}S_{W}$$ (3)

$${\dot{L}}_{D}=k_{4}DL-k_{-4}L_{D}$$ (4)

$${\dot{F}}_{W}=k_{2}FW-k_{-2}F_{W}-K_{su-in}k_{3}LF_{W}+k_{-3}L_{F}$$ (5)

L˙F=Ksu−ink3LFW−k−3LF−k5LFC−k5LFC′ (6)

$${\dot{C}}_{L}=k_{5}L_{F}C-k_{-5}C_{L}$$ (7)

C˙L′=k5LFC′−k−5CL′ (8)

$$\dot{C}=-K_{su-in}k_{5}L_{F}C+K_{su-in}k_{-5}C_{L}-k_{6}CB+k_{-6}C_{B}$$ (9)

C˙′=−Ksu−ink5LFC′+Ksu−ink−5CL′−k6′C′B+k−6CB′ (10)

B˙=k7−k8B−k6CB−k6′C′B (11)

$$C_{B}=C_{T}-C-K_{su-in}C_{L}$$ (12)

CB′=CT′−C′−Ksu−inCL′ (13)

L=LT−LF−CL−CL′−LD (14)

F=FT−FW−LF−CL−CL′ (15)

W=WT−(FW+LF+CL)⋅Ksu−ex−CL′⋅Ksu−ex−SW (16)

$$S=S_{T}-S_{W}$$ (17)

$$D=D_{T}-L_{D}\cdot K_{su-ex}$$ (18)

The modeled variables represent the following: extracellular free Wnt (W); Frizzled receptors–free (F) and bound to Wnt (F_W_); free LRP receptors (L); ternary receptor complexes Frizzled/Wnt/LRP (L_F_); intracellular destruction complex of two types resulting in different alleles–free (C, C'), bound by the ternary receptor complex (C_L_, C_L_') and bound to β-catenin (C_B_, C_B_'); intracellular free β-catenin (B); extracellular sFRP1 –free (S) and bound to Wnt (S_W_); extracellular free Dkk1 (D); and LRP receptors bound to Dkk1 (L_D_). The system is closed by seven conservation equations for the numbers of Frizzled (F_T_) and LRP (L_T_) receptors and the total concentrations of Wnt (W_T_), sFRP1 (S_T_), Dkk1 (D_T_) and destruction complex of two types (C_T_, C_T_').

Reaction rates are given by the coefficients *k*_*±i*_, where *i* is the reaction step index as shown in Fig 1, and the sign corresponds to the reaction direction. Reaction rates *k*_*6*_, *k*_*6*_*'* are different for the two complexes, as *k*_*6*_ is assumed to be affected by truncation mutations in APC. *K*_*su-ex*_ and *K*_*su-in*_, are partition coefficients translating molecule numbers in cell membrane to extracellular and intracellular concentrations, respectively. These coefficients were determined by the experiment-specific parameters *V*_*cell*_, *V*_*exp*_ and *N*_*cell*_, (cell volume, experimental well volume and number of cells per well, respectively), exactly in the same way as in [20].

The assembly of destruction complex is described by a closed equation system (19–26). In the setting of heterozygosity, two types of APC in the system (i.e., WT and a mutated APC, or two differently mutated APCs, denoted *P*, *P'*) create two types of complexes in the cell (*C*_*T*_, *C*_*T*_*'*), participating in the Wnt pathway. In such a case there are also two, possibly different, parameters for affinity of APC to Axin (denoted *K*_*D1*_, *K*_*D1*_*'*) and for the total cellular concentration of APC protein (denoted *P*_*T*_, *P*_*T*_*'*). All other parameters are assumed to be unaffected by APC mutations. The specific case of homozygous cell is represented by setting P' = A_P_' = C_T_' = 0, which effectively cancels all boxed terms and equations. In this case, the total concentration of APC protein (*P*_*T*_), and consequently, of the destruction complex (*C*_*T*_), represent the same protein/complex produced from both alleles. Parameter values for the case of WT APC can be found in Table 1.

$$\frac{P\cdot A}{A_{P}}=K_{D1}$$ (19)

$$\frac{A_{P}\cdot G}{C_{T}}=K_{D2}$$ (20)

P′⋅AAP′=KD1′ (21)

AP′⋅GCT′=KD2 (22)

P=PT−AP−(CT+CT′) (23)

P′=PT′−AP′−(CT+CT′) (24)

A=AT−(AP+AP′)−(CT+CT′) (25)

G=GT−(CT+CT′) (26)

The variables represent concentrations of free APC (*P*, *P*'), Axin (*A*) and GSK (*G*), Axin/APC dimer (*A*_*P*_, *A*_*P*_') and the destruction complex (*C*_*T*_, *C*_*T*_'). The parameters are total concentrations of the proteins (denoted by subscript “T”) and dissociation constants of APC from the Axin/APC dimer, and of the dimer from the destruction complex (*K*_*D1*_ and *K*_*D2*_, respectively).

We assumed that TCF activity is directly controlled by cellular/nuclear β-catenin level [8, 61]. In a previous work [22], Hill function was used to model the relationships between the concentration of β-catenin/TCF complex and TCF activity level (Fig 5 in [22]). Here we simplified the representation of this stage, assuming a direct relation between β-catenin accumulation and TOP/FOP reporter levels, based on the experimental data (Fig 6D in [62]). We found that this dependence can be well described by power-law:

$$\left[T\right]={\left[B_{detect}\right]}^{a}.$$ (27)

In this equation, *T* stands for TCF activity level, and *B*_*detect*_ *= (B+C*_*B*_*)* is total intracellular β-catenin concentration (*B* is intracellular free β-catenin and *C*_*B*_ is β-catenin bound to destruction complex; see S1 Text). Assuming *C*_*B*_ *<< B*, *B*_*detect*_ reflects the concentration of active β-catenin in the cell. The latter assumption is consistent with earlier observations [39], that APC levels are low, as compared to β-catenin, and is also supported by the model simulations under the estimated parameters. This assumption can be rationalized by the relatively high value of *k*_*-6*_ and the high value of dissociation constant *k*_*-6*_*/k*_*6*_, which result in low accumulation of APC/ β-catenin bound complex.

### Evaluation of model parameters

First, we estimated model parameters for the case of normal Wnt activity, based on published experimental data [31, 40]. The parameters of equation system (19–26), related to the formation of destruction complex, were estimated based on data of concentrations of the proteins composing the destruction complex, as measured in human cell lines [40]; the parameter of β-catenin–TCF correlation was calibrated based on data of both TCF activity and β-catenin accumulation as measured in cells with different mutations [32] (S2 Fig); the rest of model parameters were calibrated by simulating an experimental dataset of β-catenin accumulation under different Wnt concentrations in mouse L-cells [31], while setting some of the parameters to values taken from [20] and adjusting the others to fit the experimental results (cf. [20]). All parameter values for the case of normal cells are reported in Table 1. For details of the calibration methods, see S1 Text.

Next, we adjusted model parameters to quantitate the effect of APC mutations. The majority of APC truncation mutants lack some Axin and β-catenin binding motifs [10, 12, 49]. We assumed that when this happens, the affinity of the truncated APC to Axin is reduced, relative to WT (reflected in our model by elevated *K*_*D1*_), but that GSK binding to APC-Axin dimers is not affected, since Axin is the scaffold protein [57]. We also assumed that a destruction complex composed of truncated APC bears lower affinity to β-catenin, as compared to the WT (reduced *k*_*6*_) but not to the ternary complex, which also binds to Axin rather than directly to APC [63, 64]. In this model, reducing APC–β-catenin binding affinity affects *k*_*6*_ but not *k*_*-6*_, since the latter represents the rate of β-catenin phosphorylation, dissociation from the complex and degradation, lumped into one step. Therefore, APC truncation is represented in the model by changing the values of two of the parameters: *k*_*6*_ *≤ k*_*6WT*_ and *K*_*D1*_ *≥ K*_*D1WT*_, where *k*_*6WT*_ and *K*_*D1WT*_ are the values for WT APC (S1 Table).

We evaluated the parameters *K*_*D1*_ and *k*_*6*_ for the truncated proteins APC^1572T^ and APC^min^ (see Fig 1B). For each of these two mutants, the parameters were calibrated separately by simulating TCF activity levels in mouse ES cells carrying these mutations, and comparing to experimental data (APC^min/min^ [29], APC^1572T/1572T^ [33] and APC^1638N/1572T^ [34]; S1 Table). The calibration was performed by repetitive applications of local search algorithm *trust-region* in MATLAB, each time starting with a random initial guess for these two parameters, while the rest of parameters were set as in WT cells (Table 1). In addition, a wide range of hypothetical mutations was represented by combinations of parameter values for the truncated APC protein in the range 0.05 ≤ *K*_*D1WT*_ / *K*_*D1*_ ≤ 1, and 0.05 *≤ k*_*6*_ / *k*_*6WT*_ *≤* 1.

In all these simulations, experimental conditions were assumed to be similar to those in [32] (see Table 1). However, Wnt concentration in the experimental tissue culture systems is not reported [29, 32–34]. This is because Wnt is secreted by the co-cultured feeder cells and its concentration in the medium is not measured. In order to evaluate it, we used data of β-catenin level in cells with different mutations, neoR/neoR and neoF/neoF, in which APC protein is fully active, but its level is attenuated to 20% and 10% of the level in WT cells, respectively [32]. These data were compared to simulation results of the effect of reducing concentration of total APC in the system (*P*_*T*_), from 100% to 0% of its value in WT cells (See S3 Fig). Similar simulations were conducted with various concentrations of Wnt, while other experiment-specific parameters (i.e., volume of experiment and number of cells) were taken from [42]. The model was simulated over 7 days, to confirm that the β-catenin concentration has reached steady state. Increasing the Wnt concentration up to ~5nM caused reduction of β-catenin levels, reaching saturation at higher Wnt levels. Since simulation results at 5nM Wnt agree with the experimental data [32], we set Wnt concentration to 5nM in all further simulations.

### Simulations of inhibitory effects on mutated cells

Effects of extracellular inhibitors on TCF activity level in mutant cells were simulated setting the specific APC parameters (*P*_*T*_, *K*_*D1*_, *k*_*6*_) for the mutants. For each mutant, we simulated the effects of adding sFRP1 and Dkk1 alone or in combination, in different concentrations within the ranges: 0–500 nM for Dkk1 and 0–5000 nM for sFRP1. This was done both for the known mutants, with parameters calibrated by experimental data, and for a range of hypothetical mutations with varying parameters.

## Supporting information

S1 Fig Synergistic effects of sFRP1 and Dkk1 on APC^1638N/1572T^ mutant cells as predicted by the mathematical model. Panels (A) and (C) show model predictions for the combined inhibitory effect of sFRP1 and Dkk1 on b-catenin levels (relative to WT), with different parameter values on two edges of the biologically plausible parameter range; *k*_6_/*k*_6*WT*_ = 0.39, *K*_*D*1_/*K*_*D*1*WT*_ = 1 and *k*_6_/*k*_6*WT*_ = 1, *K*_*D*1_/*K*_*D*1*WT*_ = 4.3 in (A) and (C), respectively. The surface shade is changing with the β-catenin level. The black solid curves on the surface are contours at several fixed β-catenin accumulation levels (isoboles). Panels (B) and (D) present isobolograms (graphs of isoboles) for the combined effect of the inhibitors, simulated using the same parameters as in (A) and (C), respectively. Each curve represents all combinations of sFRP1 and Dkk1 that inhibit β-catenin accumulation to a specific level in the presence of a given Wnt3a concentration. The predicted synergism is illuminated by the convex of the curves (an additive effect would have resulted in linear curves). The black line represents effective synergistic combination of sFRP1-Dkk1, reducing β-catenin to its level in WT cells. The points denoted ‘*s*’ mark the maximally synergistic combination predicted (cf. [20]).
(TIF)

S2 Fig Power-law relationship between TCF activity and β-catenin levels. Power-law model parameter *a* was fitted to the experimental data from [32]. The black line shows the fitted curve; the dots show the WT and the experimental values of β-catenin levels and TCF activity for three different kinds of mutated cells: neoR/neoR, neoF/neoF and Δ716/Δ716 (β-catenin level is 2.6, 3.7 and 10.4 relative to its level in WT, respectively). The fit was performed using the mean values only. Error bars are reproduced from [32].
(TIF)

S3 Fig Evaluation of Wnt concentration in the tissue culture experimental systems. Simulation results for β-catenin levels at different Wnt concentrations (see box for line-types code) are presented in comparison to experimental results for cells carrying the mutations neoF/neoF and neoR/neoR. These mutants produce full-length APC, however its concentration is attenuated to 10% and 20% as compared to WT cells, respectively (red dots; data from [32]). Simulation results are in agreement with the experimental results under sufficiently large Wnt concentration (5nM). Error bars are reproduced from [32].
(TIF)

S1 Table TCF activity levels predicted for different simulated mutants. (DOCX)

S1 Text Evaluation of model parameters for Wnt pathway with non-mutated APC. (DOCX)

S2 Text Sensitivity analysis of the model. (DOCX)
