# Supplementary figures and images for: Extracellular inhibitors can attenuate tumorigenic Wnt pathway activity in adenomatous polyposis coli mutants: Predictions of a validated mathematical model
Source: PLoS One. 2017 Jul 14;12(7):e0179888. doi: 10.1371/journal.pone.0179888 (PMC5510801; doi:10.1371/journal.pone.0179888)

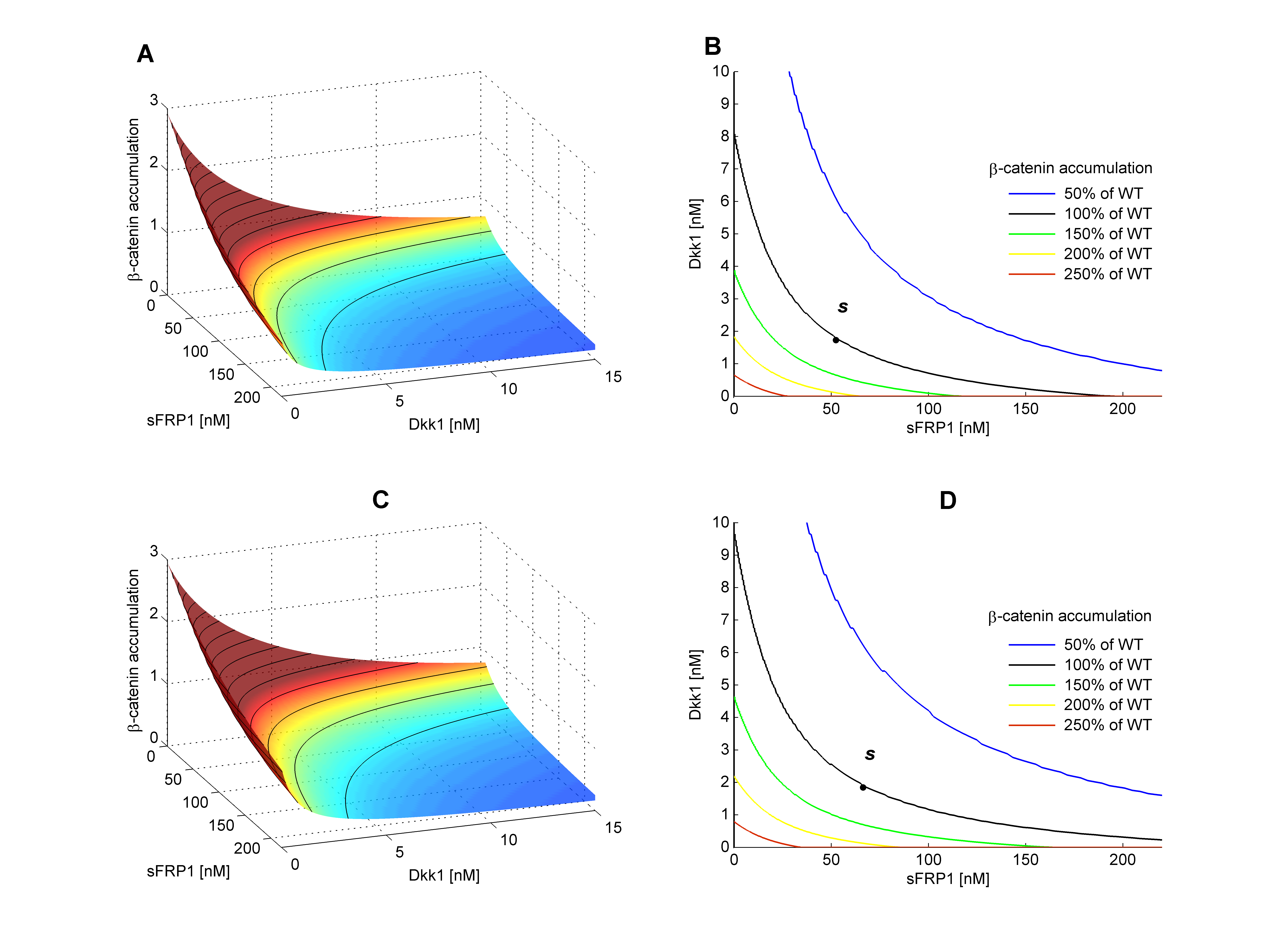

Supplement: S1 Fig — Panels (A) and (C) show model predictions for the combined inhibitory effect of sFRP1 and Dkk1 on b-catenin levels (relative to WT), with different parameter values on two edges of the biologically plausible parameter range; k6/k6WT = 0.39, KD1/KD1WT = 1 and k6/k6WT = 1, KD1/KD1WT = 4.3 in (A) and (C), respectively. The surface shade is changing with the β-catenin level. The black solid curves on the surface are contours at several fixed β-catenin accumulation levels (isoboles). Panels (B) and (D) present isobolograms (graphs of isoboles) for the combined effect of the inhibitors, simulated using the same parameters as in (A) and (C), respectively. Each curve represents all combinations of sFRP1 and Dkk1 that inhibit β-catenin accumulation to a specific level in the presence of a given Wnt3a concentration. The predicted synergism is illuminated by the convex of the curves (an additive effect would have resulted in linear curves). The black line represents effective synergistic combination of sFRP1-Dkk1, reducing β-catenin to its level in WT cells. The points denoted ‘s’ mark the maximally synergistic combination predicted (cf. [20]). (TIF) [file pone.0179888.s001.tif]

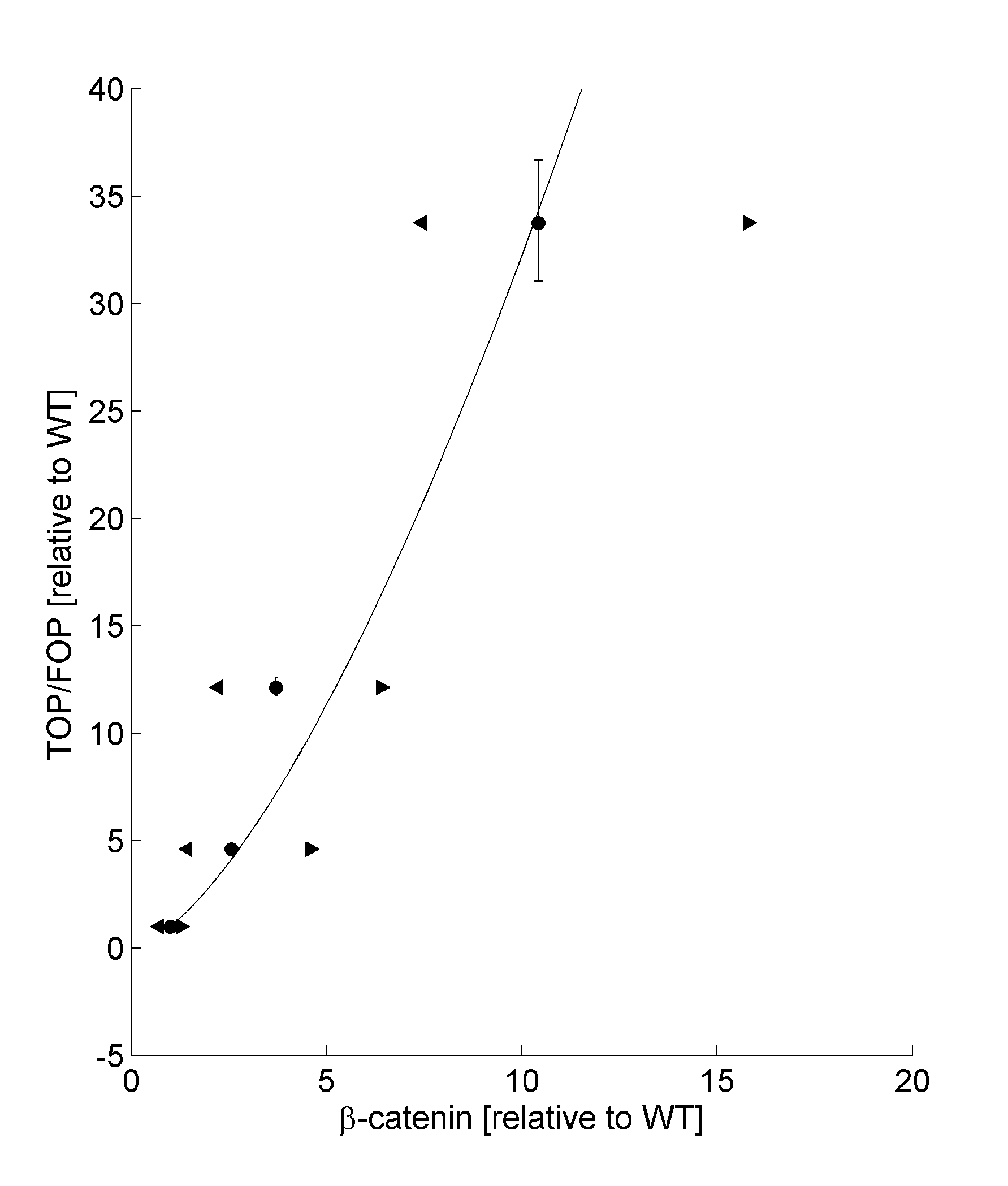

Supplement: S2 Fig — Power-law model parameter a was fitted to the experimental data from [32]. The black line shows the fitted curve; the dots show the WT and the experimental values of β-catenin levels and TCF activity for three different kinds of mutated cells: neoR/neoR, neoF/neoF and Δ716/Δ716 (β-catenin level is 2.6, 3.7 and 10.4 relative to its level in WT, respectively). The fit was performed using the mean values only. Error bars are reproduced from [32]. (TIF) [file pone.0179888.s002.tif]

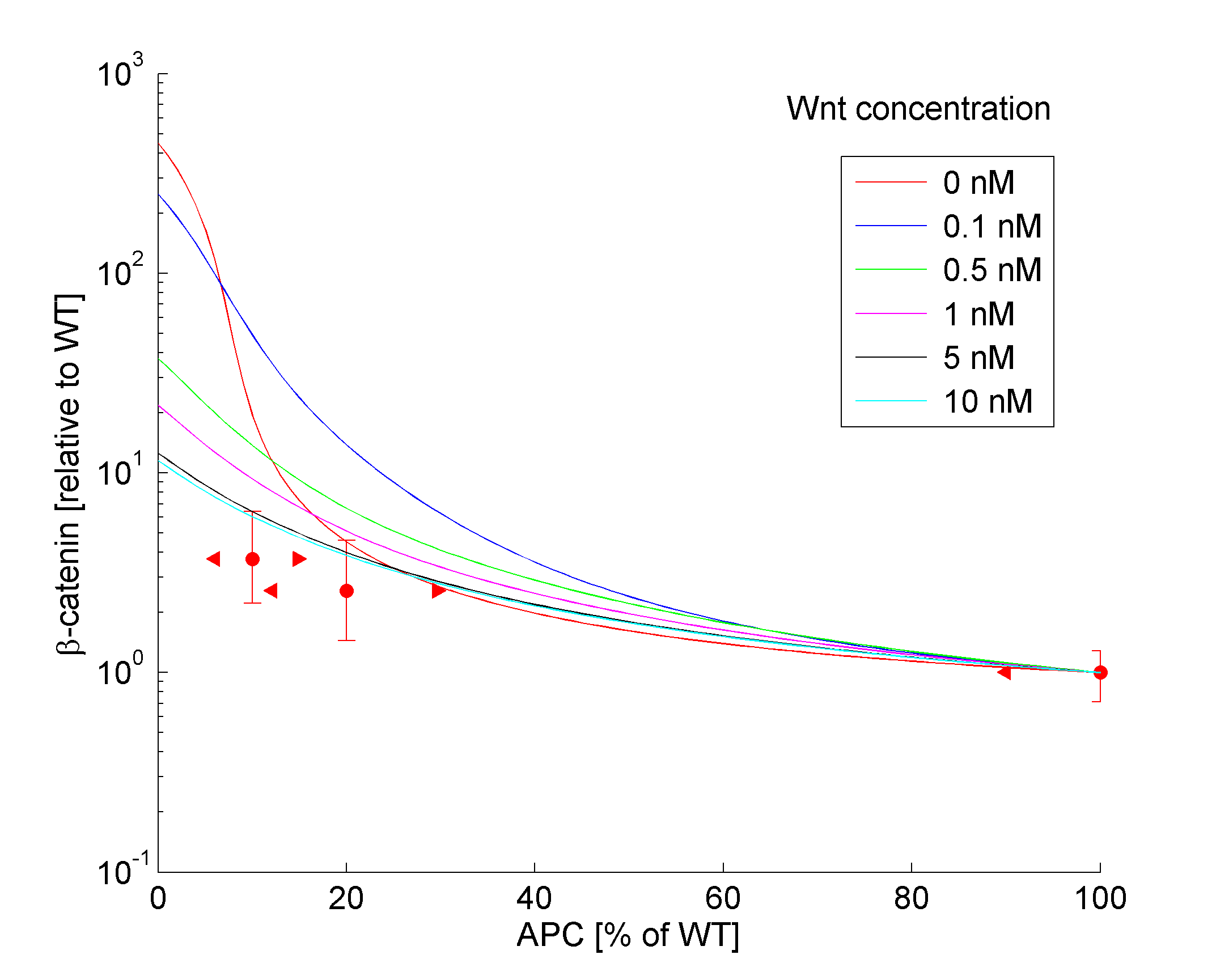

Supplement: S3 Fig — Simulation results for β-catenin levels at different Wnt concentrations (see box for line-types code) are presented in comparison to experimental results for cells carrying the mutations neoF/neoF and neoR/neoR. These mutants produce full-length APC, however its concentration is attenuated to 10% and 20% as compared to WT cells, respectively (red dots; data from [32]). Simulation results are in agreement with the experimental results under sufficiently large Wnt concentration (5nM). Error bars are reproduced from [32]. (TIF) [file pone.0179888.s003.tif]
