# Supplementary material for: Extracellular inhibitors can attenuate tumorigenic Wnt pathway activity in adenomatous polyposis coli mutants: Predictions of a validated mathematical model
Source: PLoS One. 2017 Jul 14;12(7):e0179888. doi: 10.1371/journal.pone.0179888 (PMC5510801; doi:10.1371/journal.pone.0179888)
Supplement: S1 Table — (DOCX) [file pone.0179888.s004.docx]

**S1 Table. TCF activity levels predicted for different simulated mutants**

| **Mutation** | **% of fully active APC (WT or 1638)** | **% of low-functioning APC (1572 or min)** | **TCF activity level (relative to WT)** | | **reference** |
| --- | --- | --- | --- | --- | --- |
|  |  |  | **TOP/FOP**  **simulation** | **TOP/FOP**  **experiment** |  |
| WT (+/+) | 100 | 0 | 1.0 | 1.0 | --- |
| 1638T/1638T | 100 | 0 | 1.0 | 0.97,1.55 | [1]fig 3, [2]fig 1B |
| 1638N/1638T | 51 | 0 | 2.4 | 2.3 | [1]fig 3 |
| 1572T/1572T | 0 | 100 | 2.2 | 3.1 | [2]fig 1B |
| 1638N/1572T | 1 | 50 | 4.9 | 4.5 | [1]fig 3 |
| 1638N/1638N | 2 | 0 | 36 | 29.3,20.6,9.0 | [1]fig 3, [3]fig 1D, [2]fig 1B |
| min/min | 0 | 100 | 34.5 | 34.5 | [3]fig 1D |

Results are presented in comparison to experimental values of cells harboring WT APC, APC^1638N^, APC^1572T^ or APC^min^. The mutations *Apc^min^* and *Apc^1572T^* express low-functioning truncated APC. Other mutations are assumed to express the full-length APC, though possibly at lower expression levels than in WT cells.

**References:**

1. Smits R, Kielman MF, Breukel C, Zurcher C, Neufeld K, Jagmohan-Changur S, et al. Apc1638T: a mouse model delineating critical domains of the adenomatous polyposis coli protein involved in tumorigenesis and development. Genes Dev. 1999;13(10):1309-21. Epub 1999/05/27. PubMed PMID: 10346819; PubMed Central PMCID: PMC316713.

2. Gaspar C, Franken P, Molenaar L, Breukel C, van der Valk M, Smits R, et al. A targeted constitutive mutation in the APC tumor suppressor gene underlies mammary but not intestinal tumorigenesis. PLoS Genet. 2009;5(7):e1000547. PubMed PMID: 19578404.

3. Kielman MF, Rindapaa M, Gaspar C, van Poppel N, Breukel C, van Leeuwen S, et al. Apc modulates embryonic stem-cell differentiation by controlling the dosage of beta-catenin signaling. Nat Genet. 2002;32(4):594-605. Epub 2002/11/12. doi: 10.1038/ng1045 ng1045 [pii]. PubMed PMID: 12426568.
