# Supplementary material for: Extracellular inhibitors can attenuate tumorigenic Wnt pathway activity in adenomatous polyposis coli mutants: Predictions of a validated mathematical model
Source: PLoS One. 2017 Jul 14;12(7):e0179888. doi: 10.1371/journal.pone.0179888 (PMC5510801; doi:10.1371/journal.pone.0179888)
Supplement: S1 Text — (DOCX) [file pone.0179888.s005.docx]

# S1 Text

**Extracellular Inhibitors Can Attenuate Tumorigenic Wnt Pathway Activity in Adenomatous Polyposis Coli Mutants: Predictions of a Validated Mathematical Model**

Gili Hochman^1^, Karin Halevi-Tobias^1^, Yuri Kogan^1^, Zvia Agur^1*^

^1^ Institute for Medical BioMathematics, Bene Ataroth, Israel

^*^ Corresponding author

E-mail: [agur@imbm.org](mailto:agur@imbm.org) (ZA)

# Evaluation of model parameters for Wnt pathway with non-mutated APC

***Parameters related to the formation of destruction complex***: total concentrations of Axin, APC and GSK and two dissociation constants were first reported by Lee et al, who constructed a model for the intracellular part of Wnt pathway, based on measurements done in Xenopus extracts [1]. Recently, Tan et al. have measured concentrations of Wnt pathway proteins in five human cell lines, and found that mammalian cells have lower APC levels and significantly higher Axin levels than the Xenopus extract [2]. In order to render the model adequate for describing mammalian cells, we set values of the total concentrations of these proteins to those measured in human cell lines. To calculate destruction complex concentration *C_T_*, the equation system (19–26) was solved five times, for the five reported concentrations for each of the cell lines examined in [2], while values for the two dissociation constants were set to those calculated by Lee et al (K_6_ and K_7_ in [1]). These calculations resulted in values for *C_T_* in the range of 3-10*nM*. We chose cell line SW480 [2] as a representative case, setting Axin, APC and GSK values as measured in these cells (the mutation of APC in this cell line does not affect APC expression level). In this case, *C_T_=8.05nM*, and this value was used in this work for the normal Wnt pathway model. This value for $C_{T}$ is two orders of magnitude larger than the value for total complex concentration, formerly estimated based on the Xenopus measurements [1] (see [3]).

***Parameters of the normal Wnt pathway model:*** these were calibrated by fitting the model simulations to the experimental data of β-catenin accumulation under different Wnt concentrations in mouse L-cells [4] (cf. [3]). This procedure created several alternative sets of model parameters $\left\{F_{T},k_{3},k_{6},\lambda\right\}$ and up to three additional rate constants, usually of reactions involving destruction complex (e.g., *k_5_*). Other parameters were set as in [3], with the above mentioned exception of *C_T_*. The fit was performed by repetitive applications of the local search *trust region* algorithm available through *lsqnonlin* function in MATLAB, each application beginning with a random initial guess for the values of the parameters to be calibrated.

Each one of the alternative parameter sets, comprising five or more adjusted parameters, could restore the calibration data with high accuracy (R^2^>0.97). Out of the alternative sets we selected the one whose implementation in the model predicted β-catenin intracellular levels in the range 490-1500nM, as reported for human cells [2]. The obtained set, with new values for seven parameters: $\left\{F_{T},k_{3},k_{5,}k_{6},k_{-6},k_{7},\lambda\right\}$ (see Table 1) was used in all further simulations. We validated the new model by simulating dose responses of sFRP1, sFRP2 and Dkk1, and comparing the resulting β-catenin level with published experimental data. Simulation results and goodness of fit were similar to those of our previous model (cf. [3]).

***Correlation between measured β-catenin and TCF activity levels:*** For the evaluation of the power-law rank parameter, which is assumed to define the correlation between β-catenin and TCF activity levels, we used experimental data from Li et al [5], who measured both TCF activity and β-catenin accumulation in cells with different mutations. Since both were measured relative to their level in WT cells, the power-law correlation can be rewritten as: $[T_{rel}]=[B_{rel}]^{a}$, where$B_{rel}$ and $T_{rel}$ are representing the measured β-catenin level and TCF levels, relative to their values for the reference case of a WT cell. The value for *a* was fitted to the experimental data [5] (see S2 Fig), using *trust region* algorithm available through *lsqnonlin* function in MATLAB.

**References:**

1. Lee E, Salic A, Kruger R, Heinrich R, Kirschner MW. The roles of APC and Axin derived from experimental and theoretical analysis of the Wnt pathway. PLoS Biol. 2003;1(1):E10. PubMed PMID: 14551908.

2. Tan CW, Gardiner BS, Hirokawa Y, Layton MJ, Smith DW, Burgess AW. Wnt signalling pathway parameters for mammalian cells. PLoS One. 2012;7(2):e31882. PubMed PMID: 22363759.

3. Kogan Y, Halevi-Tobias KE, Hochman G, Baczmanska AK, Leyns L, Agur Z. A new validated mathematical model of the Wnt signalling pathway predicts effective combinational therapy by sFRP and Dkk. Biochem J. 2012;444(1):115-25. Epub 2012/02/24. doi: BJ20111887 [pii]

10.1042/BJ20111887. PubMed PMID: 22356261.

4. Hannoush RN. Kinetics of Wnt-driven beta-catenin stabilization revealed by quantitative and temporal imaging. PLoS One. 2008;3(10):e3498. PubMed PMID: 18941539.

5. Li Q, Ishikawa TO, Oshima M, Taketo MM. The threshold level of adenomatous polyposis coli protein for mouse intestinal tumorigenesis. Cancer Res. 2005;65(19):8622-7. PubMed PMID: 16204028.
