# Supplementary material for: Extracellular inhibitors can attenuate tumorigenic Wnt pathway activity in adenomatous polyposis coli mutants: Predictions of a validated mathematical model
Source: PLoS One. 2017 Jul 14;12(7):e0179888. doi: 10.1371/journal.pone.0179888 (PMC5510801; doi:10.1371/journal.pone.0179888)
Supplement: S2 Text — (DOCX) [file pone.0179888.s006.docx]

# S2 Text

**Extracellular Inhibitors Can Attenuate Tumorigenic Wnt Pathway Activity in Adenomatous Polyposis Coli Mutants: Predictions of a Validated Mathematical Model**

Gili Hochman^1^, Karin Halevi-Tobias^1^, Yuri Kogan^1^, Zvia Agur^1*^

^1^ Institute for Medical BioMathematics, Bene Ataroth, Israel

^*^ Corresponding author

E-mail: [agur@imbm.org](mailto:agur@imbm.org) (ZA)

# S2 Text. Sensitivity analysis of the model

**Definition of control coefficient**

The importance of system parameters was evaluated by computing their relative contribution to the model output, namely to β-catenin levels. To this end we carried out a numeric sensitivity analysis of the model, as described below.

To evaluate how perturbation of a parameter influences model behavior, we calculated *control coefficients*, which generally express the system's response to perturbations introduced in each of the parameters [see e.g. ref 1]. For a given parameter $k_{j}$ and system state variable $x_{i}$, the control coefficient can be defined as $C_{j}^{i}=\frac{k_{j}}{x_{i}}\frac{\partial x_{i}}{\partial k_{j}}.$ Here, we used the steady-state value of free β-catenin, $B$, as the output state variable, and calculated the control coefficients for this value and all the parameters of interest (reaction rates and internal protein levels). The calculation was performed numerically, by estimating the expressions of the form $\hat{C}_{j}^{i}=\frac{k_{j}}{B}\frac{\Delta B}{\Delta k_{j}}$ , where $k_{j}$ was set at a reference value of the tested parameter (it was equal to the estimated value shown in Table 1 in the article, unless stated otherwise); $B$ is the steady state value under the reference parameter; $\Delta k_{j}=\delta{\cdot k}_{j}$, where $\delta$ranges over relative perturbation values (usually, between $-50\%$ and $+50\%$); and $\Delta B$ is the change in the steady state value of $B$ obtained from the simulation of the system under the perturbed parameter.

**Sensitivity analysis for the parameters of the wild-type system**

For the present analysis, each parameter was perturbed by decreasing or increasing its value by 50% or less (the interval $\left[ -0.5, 0.5 \right]$ for the values of $\delta$ was sampled with the step of 0.025) and the simulation was run for a time interval which was long enough to attain a steady state (35 hours). The coefficients  $\hat{C}\left( \delta\right)=\frac{1}{\delta}\frac{\Delta B}{B}$ were computed using the steady state value of $B$, for each tested parameter.

Fig. S2A shows the coefficient values obtained for all the parameters, as a function of $\delta$, where different parameters are represented by different colors. In this case, the system was simulated with $W_{T}=5nM, S_{T}=15nM, D_{T}=1nM$. As can be seen, the sign of the control coefficient remains constant for each parameter over the whole range of $\delta$, while the magnitude changes. For many of the parameters the absolute value of $\hat{C}\left( \delta\right)$ decreases when $\delta$ grows from $-0.5$ to $0.5$. Yet, for the majority of the parameters, the relative ordering of their influence remains consistent over the whole range of perturbations. Changing the doses of Wnt and of the inhibitors over the admissible ranges (reported in Table 2 in the article), including zero values, did not affect the coefficients significantly, resulting in graphs similar to Fig. S2A (not shown).

Fig. S2A suggests that the total concentration of the complex, $C_{T},$ has the largest influence on β-catenin levels, and its constituents, APC, Axin and Gsk, follow in importance (together with the binding coefficients of the destruction complex). Another summary of the same results is given in Fig. S2B, as a bar plot, where we show for each parameter the estimation of $\hat{C}\left( -0.5 \right)$,  $\hat{C}\left( 0.5 \right)$ (i.e., the two largest perturbations assessed), and the average of  $\hat{C}\left( \delta\right)$ over the small perturbation interval $-0.1<\delta<0.1$. Again, one can see that the total concentration of the destruction complex and its constituents have the maximal influence on the system output.


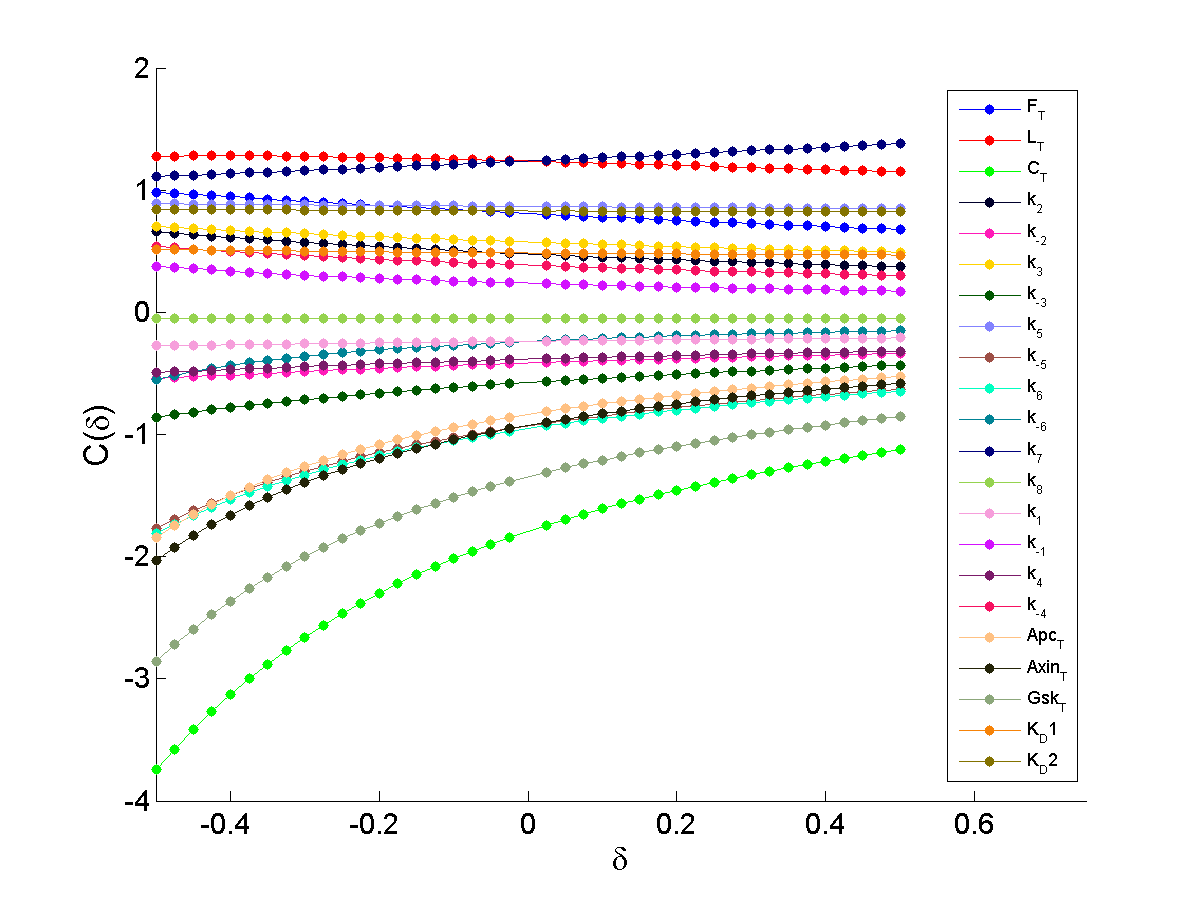


**Figure S2A.** Estimated values of control coefficients,  $\hat{C}\left( \delta\right)=\frac{1}{\delta}\frac{\Delta B}{B}$, as a function of $\delta$ for various system parameters. Each line represents the coefficient values for one parameter, color-coded as shown in the right box.


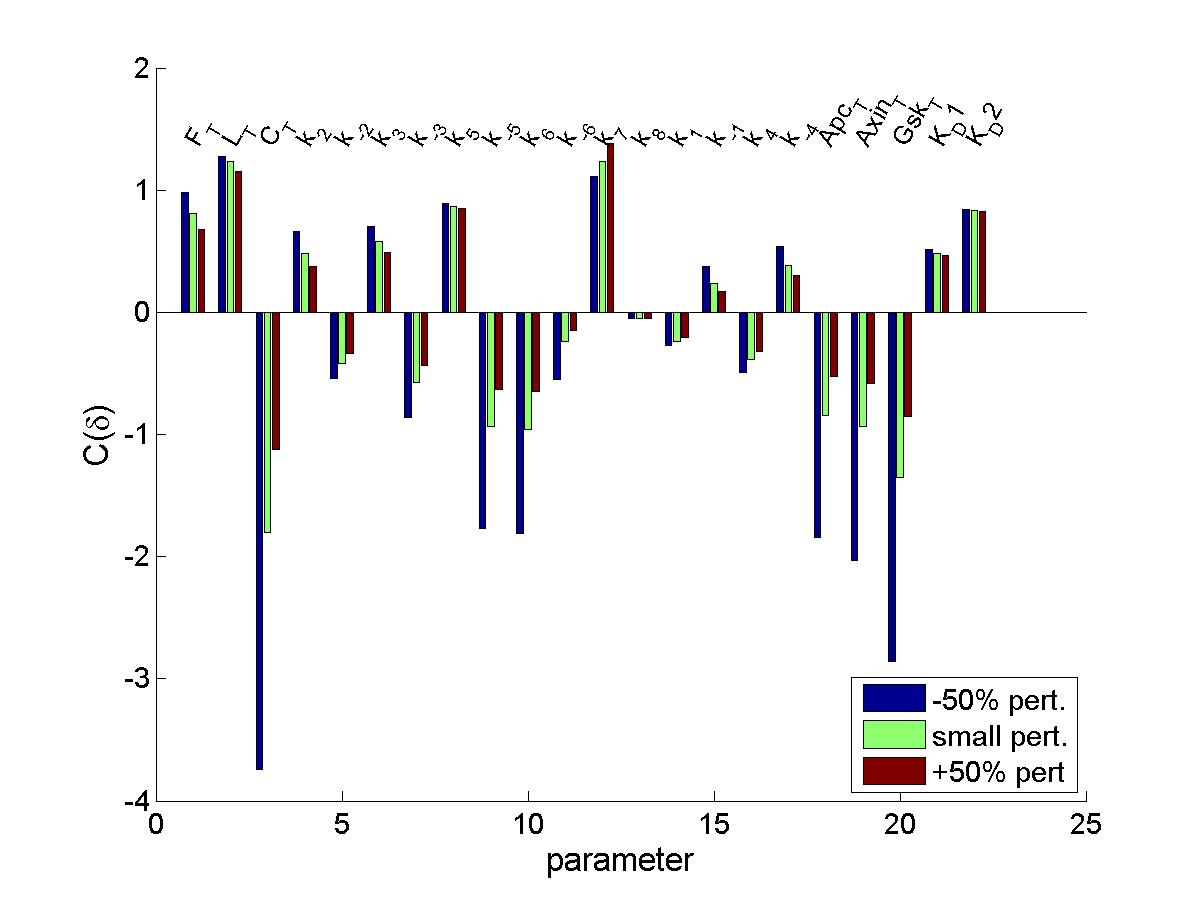


**Figure S2B.** Bar plot of control coefficients. For each parameter, designated at the top row, three histograms are shown, estimating $\hat{C}\left( 0.5 \right)$ (brown), $\hat{C}\left( -0.5 \right)$ (blue) and mean $\hat{C}\left( \delta\right)$, for $\delta\in[-0.1,0.1]$.

**Sensitivity analysis for the parameters of the mutated system**

In the main text of the article, we suggested that it is possible to keep β-catenin in check, even in the mutated cells, by increasing the concentration of the pertinent inhibitors. To apply the sensitivity analysis for supporting this finding by, we repeated the previous evaluations of the control coefficients, $\hat{C}$, for the parameters of the inhibitors binding, $k_{\pm1},k_{\pm4}$ and their concentrations $D_{T}, S_{T}$. We compared four representative parameter sets, corresponding to four of the cases in Table 3 in the main text of the article (cf. S1 Table): (1) WT parameters; (2) heterozygous APC attenuation mutation, as in APC^1638N/1638T^, reducing APC concentration to 51%; (3) homozygous APC mutation, as in APC^1572T/1572T^, reducing its binding affinity to Axin ($K_{D1})$to 20% and reducing the binding affinity of the destruction complex ($k_{6})$according to eq. (1) in the main text of the article; and (4) homozygous APC mutation$,$as in APC^min/min^ mutation also reducing $K_{D1}$ to 20%, but using eq. (2) in the main text of the article to compute the corresponding value for $k_{6}$. All other parameters and input values have been set as previously. The results are plotted in Fig. S2C (formatted similarly to Fig. S2A) in four panels relating to the abovementioned four setups. Fig. S2D shows the bar plots for these four setups, formatted similarly to Fig. S2B. It can be seen in Figs. S2C and S2D that, for mutations (2) and (3), the sensitivity to the concentrations and the reaction rates of the inhibitors remains almost unaffected, supporting the findings reported in the main text of the article. On the other hand, a strong truncation in both alleles, such as in APC^min/min^, renders the system much less sensitive to the inhibitors, due to the destruction complex being almost non-functional. This result supports our finding that mutations driving mild increase in β-catenin can be controlled by external inhibitors, while mutations that lead to high increase in β-catenin, such as APC^min/min^ are much less influenced by the inhibitors (cf. Table 3 in the main text).


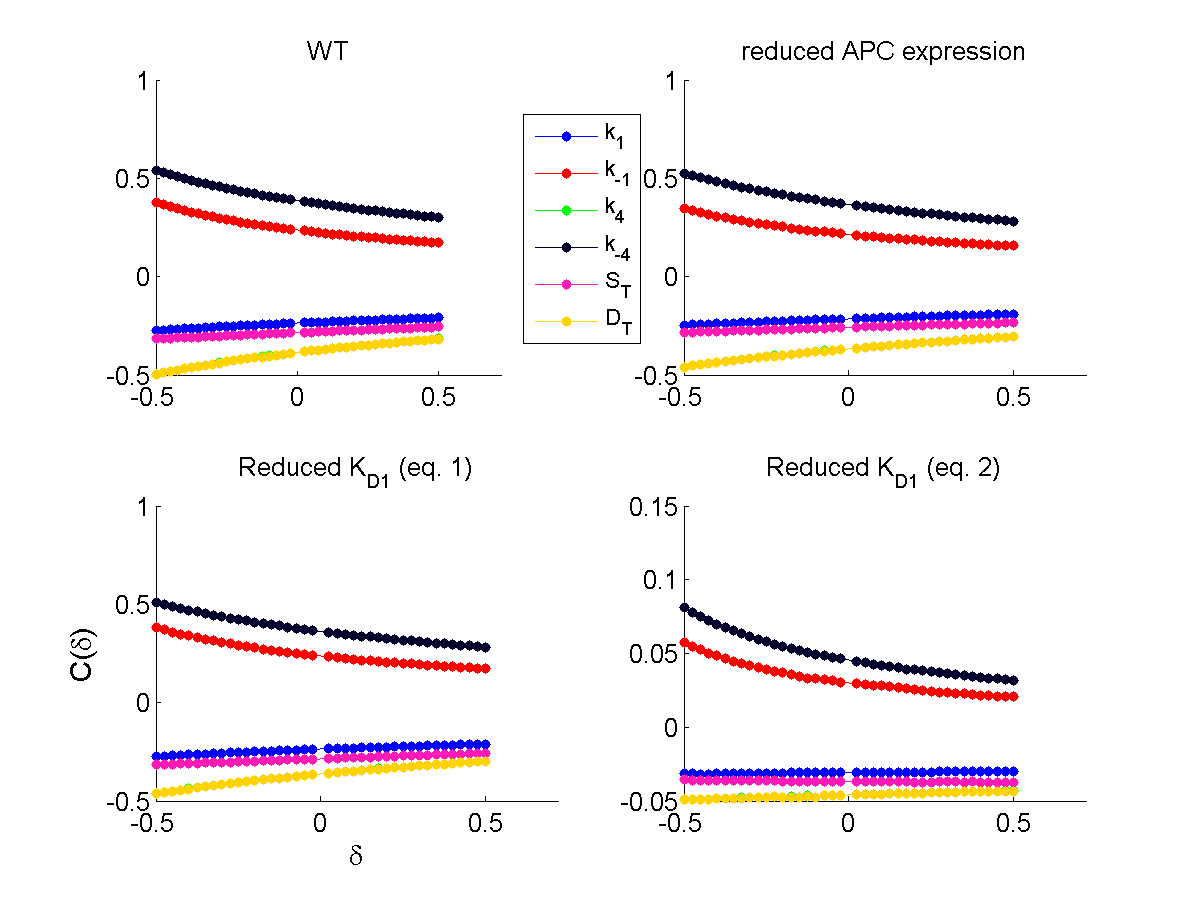


**Figure S2C.** Estimated values of the control coefficients, $\hat{C}\left( \delta\right)=\frac{1}{\delta}\frac{\Delta B}{B}$, as a function of $\delta$ for various system parameters. Each panel represents one of the four tested cases: (1) WT, (2) APC concentration reduced to 51% by the mutation, (3) $K_{D1}$ reduced to 20% by the mutation and $k_{6}$ adjusted by eq. (1) in the main text of the article, and (4) $K_{D1}$ reduced to 20% by the mutation and $k_{6}$ adjusted by eq. (2) in the article. Each line represents the results for one parameter, color-coded as shown in the right box.

**
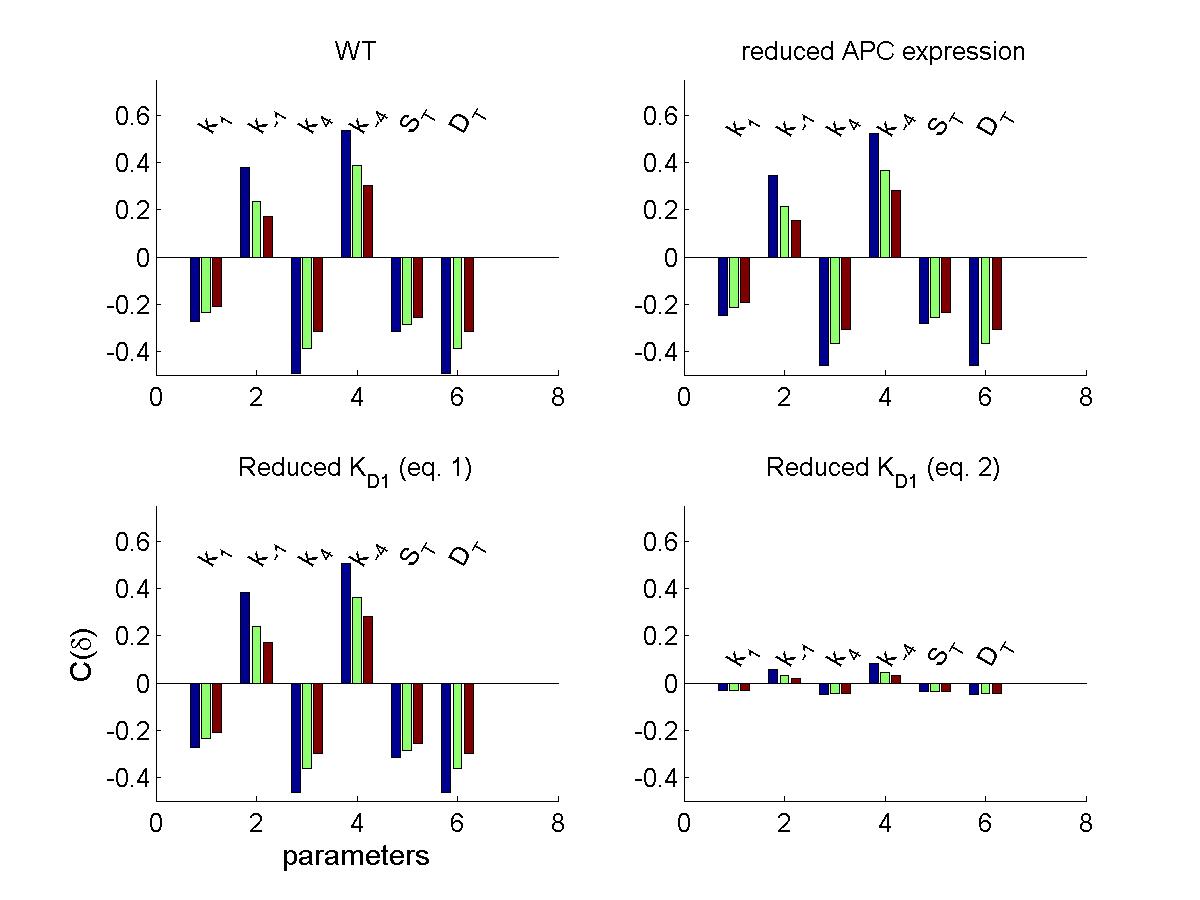
**

**Figure S2D.** Bar plot of control coefficients. Each panel represents one of the four tested cases, as in Fig. S2C. For each parameter (designated at the top), three estimations of $\hat{C}\left( \delta\right)$ are shown: $\hat{C}\left( 0.5 \right)$ (brown), $\hat{C}\left( -0.5 \right)$ (blue) and mean $\hat{C}\left( \delta\right)$, for $\delta\in[-0.1,0.1]$, as in Fig. S2B.

**References:**

1. Reinhart Heinrich, Stefan Schuster, “The Regulation of Cellular Systems”, Springer US, 1996
